# Maternal transfer of environmentally relevant polybrominated diphenyl ethers (PBDEs) produces a diabetic phenotype and disrupts glucoregulatory hormones and hepatic endocannabinoids in adult mouse female offspring

**DOI:** 10.1038/s41598-020-74853-9

**Published:** 2020-10-22

**Authors:** Elena V. Kozlova, Bhuvaneswari D. Chinthirla, Pedro A. Pérez, Nicholas V. DiPatrizio, Donovan A. Argueta, Allison L. Phillips, Heather M. Stapleton, Gwendolyn M. González, Julia M. Krum, Valeria Carrillo, Anthony E. Bishay, Karthik R. Basappa, Margarita C. Currás-Collazo

**Affiliations:** 1 Department of Molecular, Cell and Systems Biology, University of California Riverside, Riverside, CA 92521 USA; 2 Division of Biomedical Sciences, School of Medicine, University of California Riverside, Riverside, CA USA; 3 Nicholas School of the Environment, Duke University, Durham, NC USA

**Keywords:** Physiology, Diabetes, Risk factors, Diabetes

## Abstract

Polybrominated diphenyl ethers (PBDEs) are brominated flame retardant chemicals and environmental contaminants with endocrine-disrupting properties that are associated with diabetes and metabolic syndrome in humans. However, their diabetogenic actions are not completely characterized or understood. In this study, we investigated the effects of DE-71, a commercial penta-mixture of PBDEs, on glucoregulatory parameters in a perinatal exposure model using female C57Bl/6 mice. Results from in vivo glucose and insulin tolerance tests and ex vivo analyses revealed fasting hyperglycemia, glucose intolerance, reduced sensitivity and delayed glucose clearance after insulin challenge, decreased thermogenic brown adipose tissue mass, and exaggerated hepatic endocannabinoid tone in F1 offspring exposed to 0.1 mg/kg DE-71 relative to control. DE-71 effects on F0 dams were more limited indicating that indirect exposure to developing offspring is more detrimental. Other ex vivo glycemic correlates occurred more generally in exposed F0 and F1, i.e., reduced plasma insulin and altered glucoregulatory endocrines, exaggerated sympathoadrenal activity and reduced hepatic glutamate dehydrogenase enzymatic activity. Hepatic PBDE congener analysis indicated maternal transfer of BDE-28 and -153 to F1 at a collective level of 200 ng/g lipid, in range with maximum values detected in serum of human females. Given the persistent diabetogenic phenotype, especially pronounced in female offspring after developmental exposure to environmentally relevant levels of DE-71, additional animal studies should be conducted that further characterize PBDE-induced diabetic pathophysiology and identify critical developmental time windows of susceptibility. Longitudinal human studies should also be conducted to determine the risk of long-lasting metabolic consequences after maternal transfer of PBDEs during early-life development.

## Introduction

Polybrominated diphenyl ethers (PBDEs) are a class of anthropogenic persistent organic pollutants (POPs) that have been added to polymers and textiles since the 1970s to reduce the flammability of commercial products such as furniture, building materials and electronics^1^. PBDEs have been marketed as industrial mixtures, varying in percent composition of congeners based on the number of bromine substitutions on the phenyl rings, forming 209 theoretical congeners. These chemicals are lipophilic, highly resistant to degradation and are easily released from products into the indoor and outdoor environments and bioaccumulate up the human food chain^2,3^. Over a 30-year period, PBDE levels have increased exponentially in adult human tissues including blood, adipose^4^, organs^5^, breast milk, fetal^6^ and child tissues sampled worldwide^7^. Despite legislative action by the European Union and a voluntary phase-out of production in the US starting in 2005, which led to a decrease in environmental concentrations, PBDE contamination remains an ongoing problem since products containing PBDEs are still in circulation and re-entering the anthroposphere from electronic waste sites^8^ and inadvertent recycling^9^. PBDE levels in various sample types worldwide, including the breastmilk and sera of U.S. women and toddlers are still being detected^10–13^. Moreover, modelling studies predict that penta-, octa- and deca-brominated BDEs will continue to be emitted from in-use and waste stocks until 2050^9^. Given that PBDEs are still accumulating in humans, it is important to determine the long-term health consequences of chronic exposures.

Human and animal studies have associated developmental PBDE exposure with endocrine disruption, especially impaired thyroid hormone homeostasis^14,15^, neurotoxicity^15–17^ and lower birth weight and length^18^. Stressful physiological conditions during development can cause a predisposition to chronic disease later in life^19^. Such suboptimal conditions during perinatal life include exposure to PBDEs due to mobilization from maternal sources through cord blood *in utero*^20^ and breast milk during lactation^11,21^. After birth, toddlers continue to be exposed to environmental PBDEs through household dust and diet. These factors, as well as immature detoxification in the liver, contribute to a three- to nine-fold higher PBDE body burden in infants and children as compared to adults^22,23^. Thus, developmental exposure to highly penetrant and bioactive environmental xenobiotics such as PBDEs is of high concern due to potential significant health risks posed during developmental time points sensitive to biological reprogramming.

Type 2 diabetes (T2D) has shown a dramatic rise in incidence in recent years. It is estimated to become the greatest epidemic of the twenty-first century; with a predicted increase to 592 million affected by 2035^24^. Epidemiological studies suggest that the escalating production and environmental presence of a number of metabolic disrupting chemicals (MDC) over the past four decades may contribute to the pathogenesis of metabolic diseases^25^. Mounting evidence has implicated brominated POPs in the pathogenesis of T2D and metabolic syndrome (MetS)^26–34^. In particular, diabetes and/or MetS are positively associated with high body burdens of individual PBDE congeners including: BDE-28, -153, and -47, one of the most abundant PBDE congeners detected in the environment and in human tissues^29,34–36^. Additionally, exposure to BDE-28, -47, -99, -153, -154 and -183 increases the risk of gestational diabetes mellitus (GDM) in healthy US women^37–39^, a physiologically demanding condition that may increase the risk of MetS in adult offspring^40^. All of the PBDE congeners that have been associated with T2D are found in DE-71, a commercial mixture of PBDEs with high environmental and human relevance^41^. DE-71 congeners are shown to accumulate in liver and adipose tissue^17^, which are critical for glucose homeostasis.

Given that developing organisms are more susceptible to potential metabolic disruptors such as PBDEs, it is important to test whether they predispose offspring to diabetes in adulthood. To answer this question, we employed a mouse model of chronic, low-dose maternal transfer of environmentally relevant PBDE congeners to characterize the *adult* consequences of exposure that occurred during critical perinatal developmental windows. We tested the hypotheses that exposure to DE-71 produces a diabetogenic phenotype and that DE-71 effects are more pronounced in developmentally exposed female offspring versus their adult-exposed mothers. We focused on female mice, since impaired glucose tolerance is more common in diabetic women who also have an increased risk of death compared to non-diabetics^42^. Our results indicate that exposure to DE-71, especially when administered perinatally, alters clinically relevant diabetic biomarkers, namely, fasting blood glucose, glucose tolerance, insulin sensitivity, plasma levels of glucoregulatory hormones as well as liver endocannabinoid tone, an emerging biomarker of energy balance. These findings raise concern for the health of progeny of directly exposed mothers, especially since the diabetogenic effects of DE-71 involve multiple organ system biomarkers and persist into adulthood.

## Results

**Figure 1:**
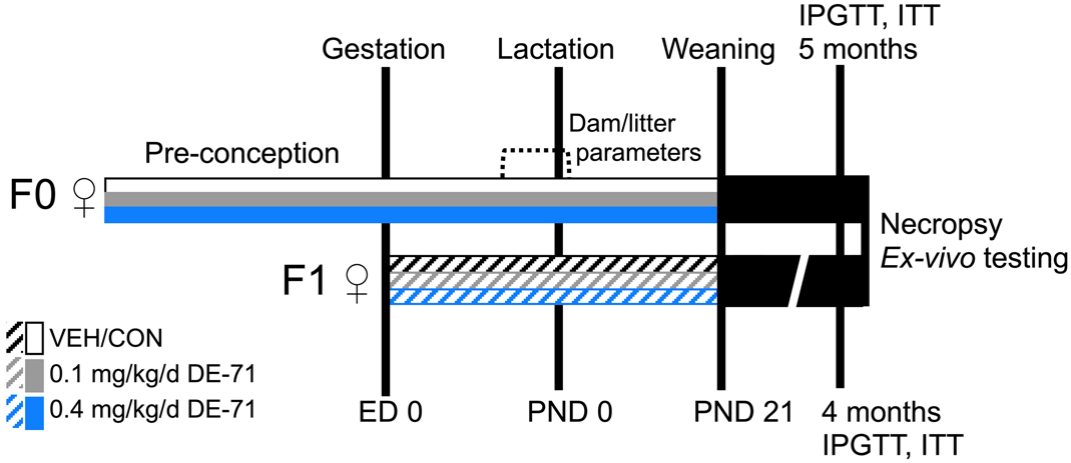
Dosing and testing paradigm used for perinatal and adult exposure to DE-71. Direct exposure to DE-71, represented by solid shading in adult dams (F0♀), began ~ 4 weeks pre-conception and continued daily for ~ 3 weeks of gestation and 3 weeks of lactation until pup weaning at PND 21. Indirect exposure to DE-71, represented by hatched shading, in female offspring (F1♀) occurred perinatally (ED 0 to GD 18) and postnatally via lactation through weaning (PND 0-PND 21) for ~ 39 days. Maternal and litter parameters were taken during GD 15 to PND 0. Using this regimen, exposure duration was 70–80 days for F0 and 39 d for F1. Metabolic endpoints (fasting glycemia, IPGTT, ITT) were examined in adulthood, at 1–2 weeks post-weaning for dams (at ~ 5 months old) and ~ 4 months for offspring. At necropsy, body and organ weights were recorded and blood and organ tissues collected for further analysis: GC/ECNI/MS BDE congener determination, plasma ELISA, UPLC/MS/MS detection of endocannabinoids, epinephrine assay, liver enzymatic activity. *GD* gestational day, *PND* postnatal day, *ED* embryonic day. This figure was created by E.V.K. using Microsoft PowerPoint for Mac Version 16.27 (https://www.microsoft.com/en-us/microsoft-365/p/powerpoint).

C57Bl/6 mouse dams were exposed to DE-71 and later investigated along with their offspring as shown (Fig. 1).

### Dam gestational parameters and litter outcomes

DE-71 exposure did not significantly affect maternal food intake, gestational weight gain, litter size or secondary sex ratio (see Supplementary Table S1 online).

### Congener analysis in livers of F0 and F1 females

**Figure 2:**
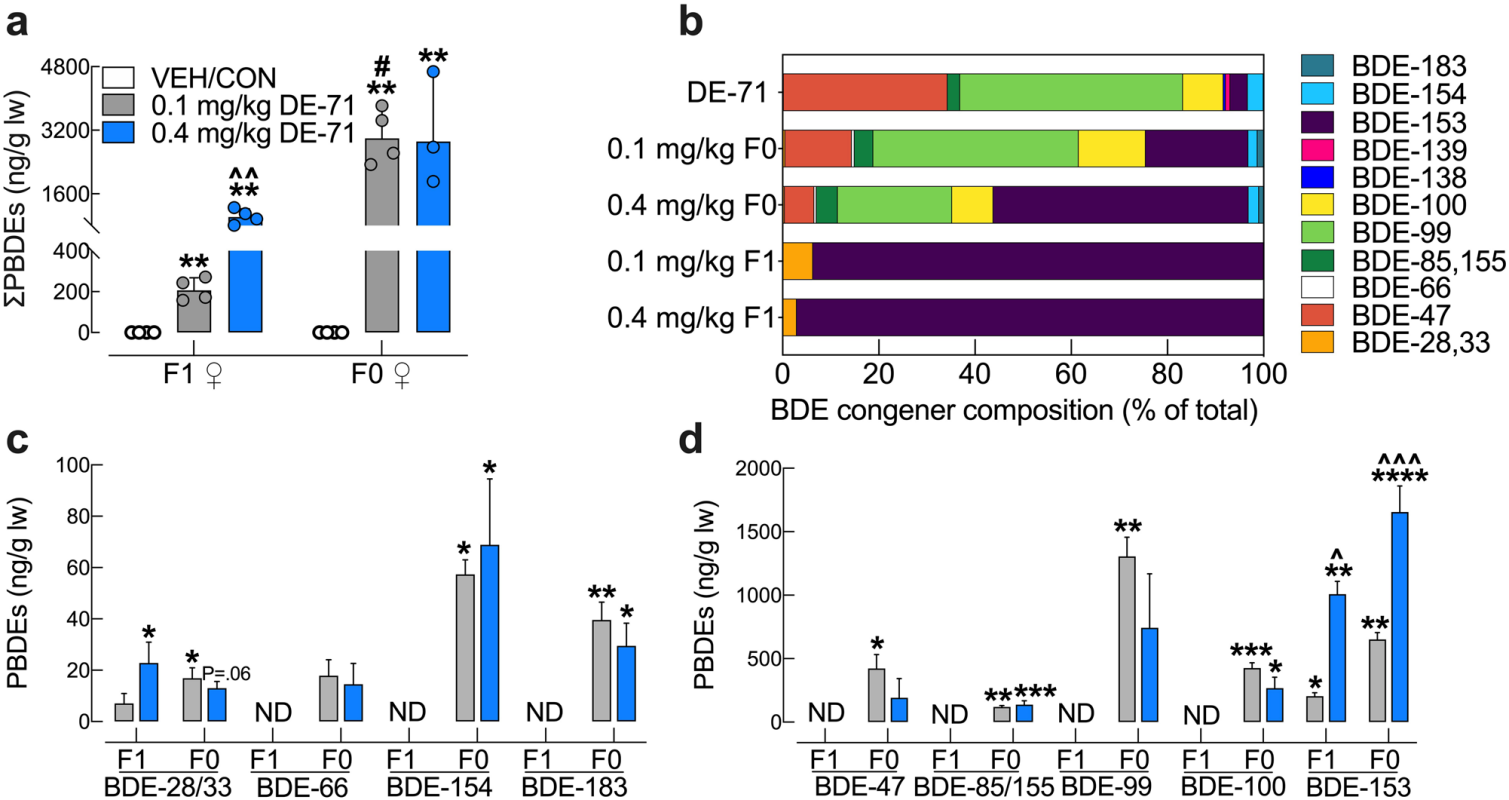
Hepatic BDE congener analysis of DE-71-exposed mouse dams and their adult female offspring. (**a**) The ng/g lipid wt sum concentrations of the PBDE congeners detected (bars represent ∑PBDEs; geometric mean ± geometric SD) were comprised of BDE 28/33, BDE-153 for F1 and BDE-28/33, BDE-47, BDE-66, BDE-85/155, BDE-99, BDE-100, BDE-153, BDE-154, BDE-183 for F0. DE-71 exposure produced significant accumulation of PBDEs in liver, being greater in directly exposed F0 versus indirectly exposed F1 female mice. Dose-dependency was only seen in F1. Values for VEH/CON are not shown since they were below the method detection limit (MDL). (**b**) BDE composition (percent total) in the lot of DE-71 used and in livers of F1 and F0. The multi-congener profile in F0 was similar to that of DE-71, whereas that of F1 was restricted to BDE-28/33 and BDE-153. Co-elution of BDE -28 and -33 as well as BDE-85 and -155 prevented differentiation during analysis. (**c**,**d**) Absolute concentrations of congeners found in F1 and F0 liver. Predominant congeners in F0 were BDE-47, -99, -100, and -153. Predominant congeners in F1 were BDE-28/33 and -153. Only BDE-153 showed a rise in content in F0 and F1 mice exposed to 0.4 mg/kg relative to 0.1 mg/kg. For statistical purposes, values below the MDL were substituted with randomly generated values between 0 and MDL/2 and designated as not detected (ND). Bars and error bars reflect mean ± s.e.m. *Indicates significantly different from VEH/CON (*P < .05, **P < .01, ***P < .001, ****P < .0001); ^indicates significantly different from the corresponding 0.1 mg/kg group (^*P* < .05, ^^*P* < .01, ^^^*P* < .001). #significant difference across F0 and F1. Dunnett’s T3 or Tukey’s post-hoc tests were used. n = 3–4 replicates/group, analyzed in triplicate. *F1* female offspring, *F0* dams, *ND* not detected.

Notable differences were observed in hepatic BDE congener accumulation after direct versus indirect exposure to DE-71 in F0 and F1 females, respectively. The sum of detected BDE congeners (∑PBDEs, geometric mean ± SD) in dams was approximately 15-fold greater relative to offspring exposed to 0.1 mg/kg DE-71 (Fig. 2a; *P* < 0.05). An effect of increasing dose was seen only in F1. Individual congener analysis indicates only tri- (BDE-28/33) and hexa-brominated (BDE-153) congeners were detected in offspring liver. In contrast, more congeners were detected in dams: tri- (BDE-28/33), tetra- (BDE-47, -66), penta- (BDE-85, -99, -100), hexa- (BDE-153, -154) and hepta-brominated congeners (BDE-183) (Fig. 2c,d). Figure 2b shows that, of the total congeners measured, the predominant ones in the dam liver were (% in 0.1–% in 0.4 mg/kg): BDE-99 (24–43%) and BDE-153 (21–53%). The overall congener pattern observed in dam liver was similar to that in DE-71^17,41^ except for the greater content of BDE-153 and the lower content of BDE-47 (6–14%) and BDE-100 (8.5–14%). In combination, these four congeners accounted for 92% (0.1 mg/kg) and 91.5% (0.4 mg/kg) of total BDEs in exposed F0 liver. In contrast, BDE-153 accounted for 97% and 94% of total PBDEs detected in F1 offspring exposed to 0.1 mg/kg and 0.4 mg/kg doses, respectively. The exaggerated penetration of BDE-153 (4 to 10-fold greater in F0 and 17 to 18-fold greater in F1 than of the congener profile of DE-71) is consistent with previous reports^43^. For complete congener profiles see Supplementary Table S2 online.

### Chronic low dose DE-71 exposure has minimal effects on body and selected organ weights

Body weights of female offspring perinatally exposed to 0.1 mg/kg were significantly lower by approximately 7% relative to VEH/CON (see Supplementary Table S3 online). Absolute liver weight was greater in 0.4 mg/kg offspring relative to VEH/CON (9%) and 0.1 mg/kg (13%). The absolute and relative weights of pancreas and spleen of the F1 females were similar across groups. The only difference seen in dams was the 9% greater relative liver weight of 0.1 mg/kg dams compared to VEH/CON. Fasting body weights taken from a subset of mice used for IPGTT and ITT were not different across groups and, therefore, the diabetogenic phenotype of DE-71-exposed F1 mice is not due to obesity.

### DE-71 produces fasting hyperglycemia in F1 but not F0 females

**Figure 3:**
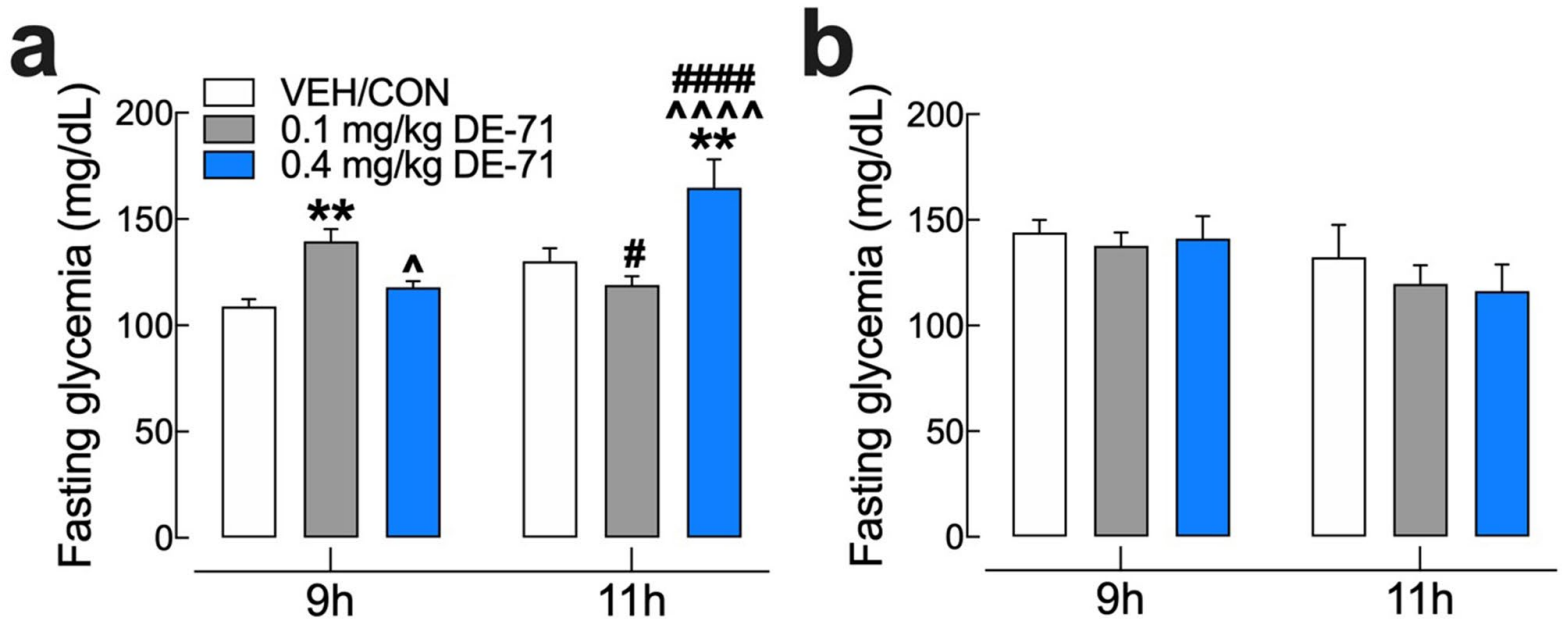
DE-71 exposure produces elevated fasting blood glucose (FBG) in perinatally exposed female offspring but not their mothers. FBG was measured after a 9 or 11 h fast in female offspring (**a**) and dams (**b**). *indicates significantly different from VEH/CON (**P < .01); ^indicates significantly different from 0.1 mg/kg DE-71 (^*P* < .05, ^^^^*P* < .0001). ^#^Indicates significant difference between 9 and 11 h for corresponding exposure group (#*P* < *.05*; ####*P* < .0001). Sidak’s post hoc test was used. Bars and error bars represent mean ± s.e.m. n = 7–12/group. *F1* female offspring, *F0* dams, *FBG* fasting blood glucose.

One indication of pre-diabetes is abnormally high fasting blood glucose (FBG) concentration^44,45^. We examined FBG after 9 and 11 h fasting using glycemia values from basal time points obtained in IPGTT and ITT experiments. For the ITT, we used a 9 h fast time and a corresponding low insulin dose (0.5 U/kg). In female offspring, 0.1 mg/kg DE-71 significantly elevated FBG after a 9 h fast and 0.4 mg/kg DE-71 elevated FBG after an 11 h fast relative to VEH/CON (Fig. 3a). Therefore, hyperglycemia was present in exposed F1 at both fast times, albeit the effective DE-71 dose was different depending on fasting duration. In contrast, DE-71 exposure did not significantly affect FBG in F0 females, regardless of the fasting condition (Fig. 3b). These results suggest that perinatal exposure to DE-71 produces fasting hyperglycemia, a condition that may be due to dysregulated endocrine parameters of glucose metabolism^46^.

### DE-71 exposure impairs glucose tolerance in F1 and F0

**Figure 4:**
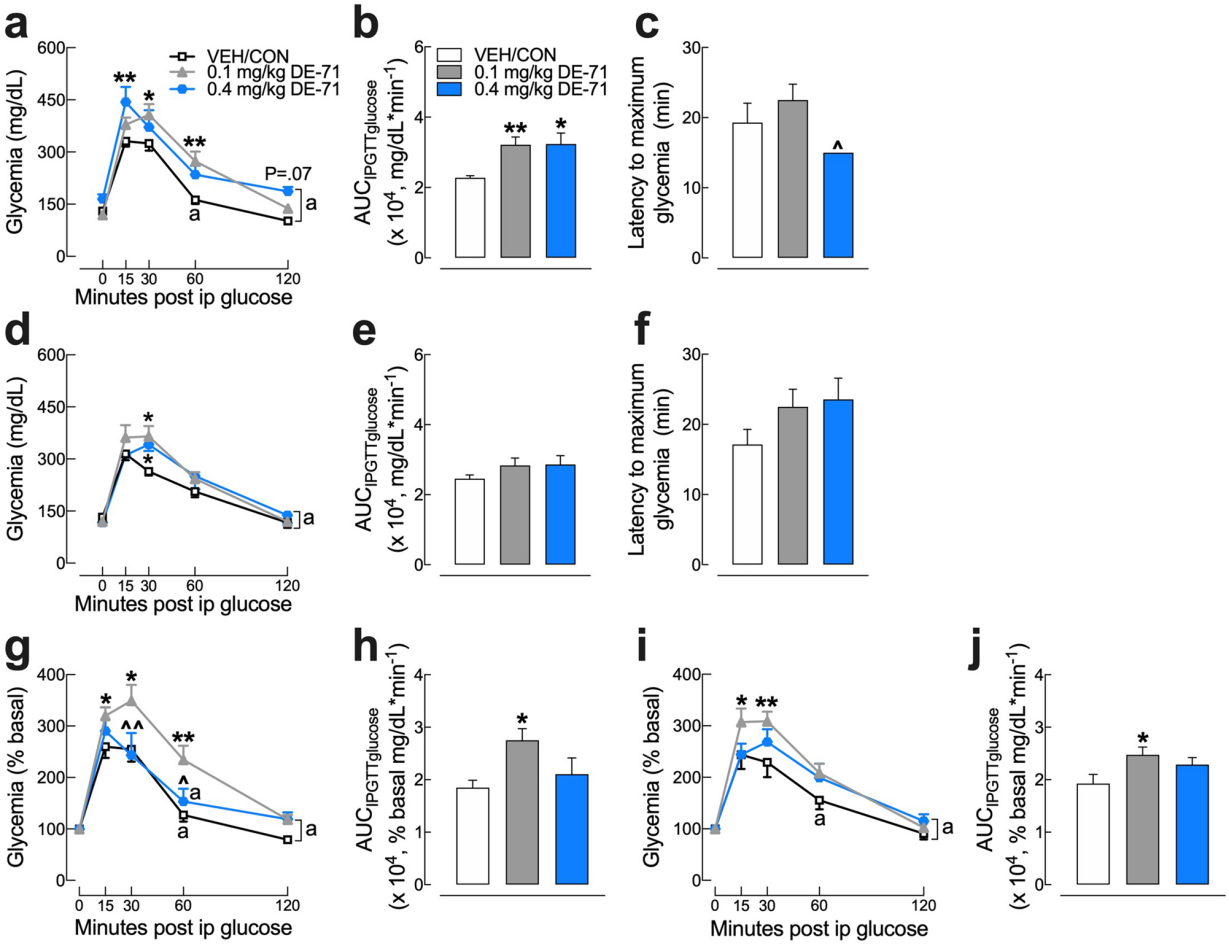
DE-71 exposure produces greater glucose intolerance after perinatal exposure compared to adult exposure. Mice were fasted for 11 h ON and tail blood was sampled for glucose before (t = 0 min) and after (t = 15, 30, 60 and 120 min) i.p. injection of 2.0 g/kg glucose. Absolute blood glucose concentrations taken during IPGTT of F1 (**a**) and F0 (**d**). Mean values for the integrated area under the IPGTT glucose curve (AUC_IPGTTglucose_) show increased glycemia for F1 (**b**) but not F0 (**e**). Blood glucose values taken during IPGTT are plotted versus time as a percent of basal glucose for F1 (**g**) and F0 (**i**). Mean values for integrated area under the IPGTT glucose percent basal curve (AUC_IPGTTglucose_) show increased glycemia for both F1 (**h**) and F0 (**j**). Latency to maximum glycemia for F1 (**c**) and F0 (**f**). *Indicates significantly different from VEH/CON (*P < .05, **P < .01); ^indicates significantly different from 0.1 mg/kg DE-71 (^*P* < .05, ^^*P* < .01). Tukey’s or Dunnett’s post hoc tests were used. The symbol “a” indicates the time points at which glycemia is not different from basal in the corresponding treatment group. Glycemia at all other time points differs from basal. Bars and error bars represent mean ± s.e.m. n = 7–12/group. *F1* female offspring, *F0* dams.

To investigate the effects of DE-71 on glucose tolerance, glycemia was measured during IPGTT. Blood glucose levels rose rapidly and peaked within 15 min of glucose challenge in the VEH/CON and the 0.4 mg/kg DE-71 groups. In contrast, the corresponding peak for the 0.1 mg/kg DE-71 group occurred later, at 30 min (Fig. 4a). Relative to VEH/CON, the glycemia was exaggerated in exposed F1 at 30 and 60 min (0.1 mg/kg) and at 15 min post injection (0.4 mg/kg) indicating glucose intolerance, with an especially pronounced magnitude in the 0.1 mg/kg DE-71 group (Fig. 4a). Plasma glucose showed a gradual return to baseline at 60 min post injection in VEH/CON. In contrast, for both exposed F1 groups, the corresponding time was 120 min, or 1 h longer (Fig. 4a). The differences in magnitude and duration of glycemia are incorporated in the area under the glucose curve, AUC_IPGTTglucose_, which is abnormally large in F1 females exposed to either dose (Fig. 4b). The latency to maximum glycemia was not significantly different across groups (Fig. 4c). Because FBG after an 11 h fast was elevated in F1 exposed to 0.4 mg/kg, glycemia, values are also normalized using percent baseline (Fig. 4g). In this case, similar results were found to those expressed using absolute glycemia values but only the 0.1 mg/kg dose group shows significantly greater AUC_IPGTTglucose_ (Fig. 4h).

Exposed F0 also showed significantly greater glycemia relative to VEH/CON but the difference was moderate and this occurred at 30 min post-injection (Fig. 4d). This was not reflected in the AUC_IPGTTglucose_ (Fig. 4e). The latency to maximum glycemia was not significantly different across treatment groups (Fig. 4f). Glycemia levels (expressed as percent of basal) returned to normal by 60 min in VEH/CON but not until 120 min in F0 exposed to either dose (Fig. 4i). In addition, F0 exposed to 0.1 mg/kg showed a significantly greater AUC_IPGTTglucose_ relative to VEH/CON (Fig. 4j). To test the hypothesis that DE-71-provoked glucose intolerance is exaggerated in F1 relative to F0, we compared percent basal AUC_IPGTTglucose_ for the 0.1 mg/kg exposure groups and found no significant differences (*P* = 0.17). These results suggest that exposure to DE-71 causes glucose intolerance after either developmental or adult exposure.

### DE-71 exposure produces an abnormal glycemic response to insulin in F1 females

**Figure 5:**
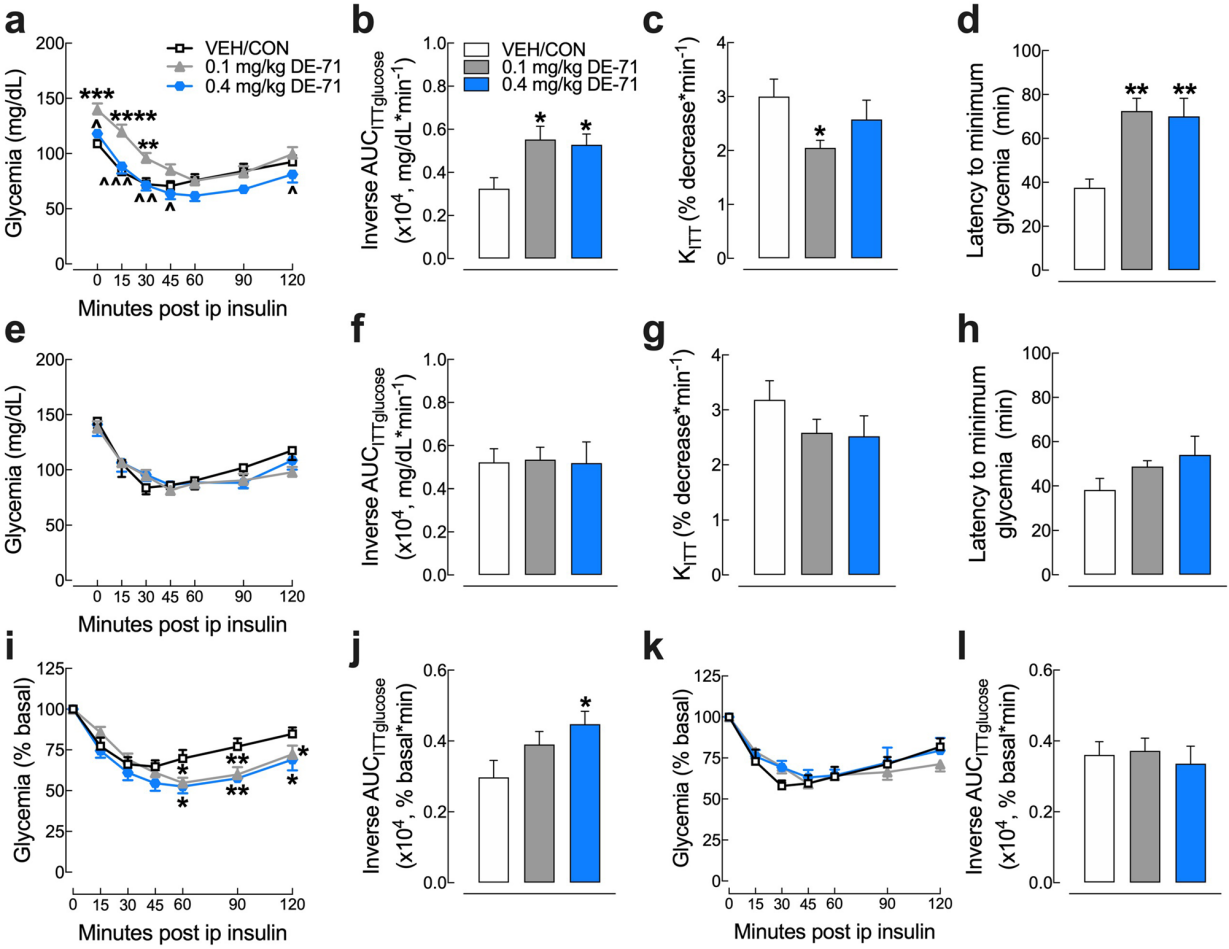
DE-71 exposure causes less glycemia reduction and delayed glucose clearance after insulin challenge in F1 but not F0 female mice. Absolute blood glucose concentrations were recorded before and at *t* = 15, 30, 45, 60, 90 and 120 min post-injection with 0.5 U/kg insulin for female offspring (F1) (**a**) and their mothers (F0) (**e**). Glycemia was analyzed by inverse integrated area under the ITT glucose curve (AUC_ITTglucose_) for F1 (**b**) and F0 (**f**). Rate constant for glucose reduction (K_ITT_) was calculated over the initial slope of ITT glucose response curve from 0–15 min post-injection and was significantly reduced for F1 at 0.1 mg.kg (**c**) but not for F0 (**g**). Latency to minimum blood glucose measured over the two hour time course of ITT glucose response curve for F1 (**d**) and F0 (**h**). Glucose values taken during ITT are plotted versus time as a percent of the individual baseline for F1 (**i**) and F0 (**k**). The inverse integrated area (AUC) under the percent basal glucose curve (AUC_ITTglucose_) shows a significant increase commensurate with a prolonged insulin response for F1 (**j**) but not F0 (**l**). *Indicates significantly different from VEH/CON (*P < .05; **P < .01, ***P < .001, ****P < .0001). ^Indicates significantly different from 0.1 mg/kg DE-71 (^*P* < 0.05, ^^*P* < .01, ^^^*P* < .001). Glycemia at all time points differs from baseline for corresponding group. Dunnett’s and Tukey’s *post-hoc* tests were used. All values represent mean ± s.e.m. n = 8–12/group. *F1* female offspring, *F0* dams.

Next, we examined the glycemia response to exogenous insulin during ITT experiments. Mean glycemia values over the 120 min period following insulin injection are shown in the insulin tolerance curve (Fig. 5a). F1 exposed to 0.1 mg/kg DE-71 display less reduction in glycemia as compared to VEH/CON at several time points post injection (*t* = 15, 30 min). However, this was confounded by the elevated FBG for F1 exposed to 0.1 mg/kg (Fig. 3a). Therefore, a more valid group comparison is shown when expressing glycemia as a percent of baseline (Fig. 5i). In this case, exposed F1 displayed a deeper insulin curve with a longer recovery time after insulin injection. This is represented as a greater mean latency to reach the minimum insulin-induced hypoglycemia in exposed F1 groups, 72.5 min (0.1 mg/kg) and 70 min (0.4 mg/kg), relative to VEH/CON, i.e., 37.5 min (Fig. 5d), possibly indicating delayed glucose clearance/utilization in response to insulin (Fig. 5d).

The inverse area under the glucose response curve showed a greater insulin-induced glucose response for F1 exposed to either DE-71 dose (Fig. 5b). In contrast, the inverse AUC_ITTglucose_ using percent baseline glycemia showed a significant increase only for the 0.4 mg/kg exposed F1 group relative to VEH/CON (Fig. 5j). Because the glycemia response over the 120 min observation period is due to complex actions of insulin (insulin signaling at its targets-sensitivity, half-life, and glucose utilization/clearance), we measured early effects of insulin (sensitivity), represented as K_ITTinsulin_ measured over the first 15 min post-injection (Fig. 5c). This metric showed a significant decrease (32%) in blood glucose reduction rate for 0.1 mg/kg F1 (*P* = 0.04) although not 0.4 mg/kg F1 (14%) relative to VEH/CON, suggesting that DE-71 exposure at 0.1 mg/kg produces significant insulin insensitivity.

In contrast to that of F1, the ITT curve for exposed F0 appears normal (Fig. 5e,k). No statistically significant group differences for F0 were observed for K_ITTinsulin_ (Fig. 5g). The mean decrease in K_ITTinsulin_ values relative to VEH/CON was 19 and 21% greater for F0 exposed to 0.1 and 0.4 mg/kg DE-71, respectively. In addition, exposed F0 showed normal latency to reach minimum insulin-induced hypoglycemia relative to VEH/CON (Fig. 5h). Accordingly, no group differences in F0 were seen for inverse AUC_ITTglucose_ (Fig. 5f,l). Taken together, these results indicate reduced insulin sensitivity (0.1 mg/kg) and delayed glucose clearance/utilization (0.1 and 0.4 mg/kg) in response to exogenous insulin injection in exposed F1 but not F0. These results confirm our hypotheses that female offspring of dams exposed to DE-71 during pregnancy are more susceptible to insulin insensitivity characterized by a delay in reaching a peak response to and delayed recovery from insulin challenge, suggesting a diabetogenic phenotype. Whether exposure to DE-71 in dams produced gestational diabetes followed by a normalization in insulin sensitivity after pregnancy when dams were tested is unclear.

### Endocrine-disrupting effects of DE-71 exposure on glucoregulatory hormones

**Figure 6:**
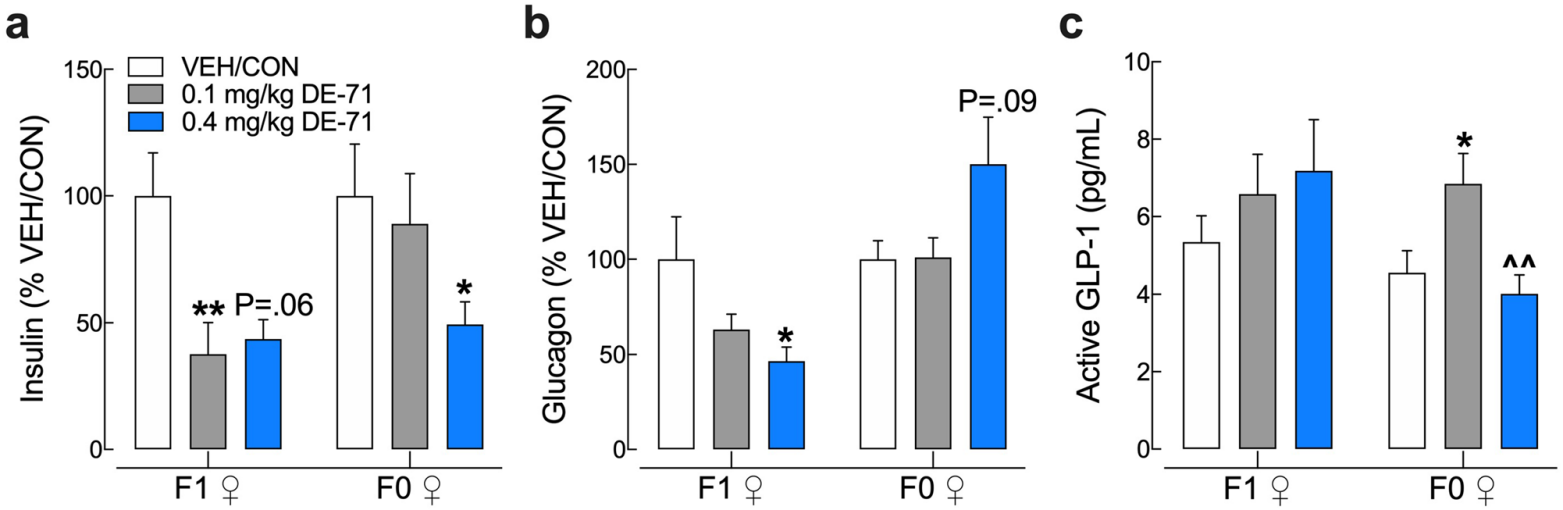
Endocrine-disrupting effects of DE-71 on glucoregulatory hormones in F0 and F1 female mice. Blood collected at sacrifice was assayed for plasma levels of insulin (**a**), glucagon (**b**), and GLP-1 (**c**) in offspring and dams using specific EIA. F1 exposed to DE-71 displayed reduced insulin (1.0 mg/kg) and an apparent reduction in insulin and reduced glucagon (0.4 mg/kg). F0 exposed to DE-71 displayed reduced plasma insulin and apparent rise in glucagon (0.4 mg/kg). *Indicate significantly different from corresponding VEH/CON (*P < .05, **P < .01). ^Indicates significantly different from 0.1 mg/kg DE-71 (^^*P* < .01). Bars and error bars represent mean ± s.e.m. Insulin and glucagon levels are expressed as a percent of VEH/CON. Dunnett’s T3 Dunn’s and Tukey’s post hoc tests were used. n = 12–24/group for insulin, n = 6–8/group for glucagon and n = 8–14/group for GLP-1. *EIA* enzyme-linked immunoassay, *F1* female offspring, *F0* dams.

Having observed disruptions in glucose homeostasis we measured plasma hormones involved in carbohydrate regulation using EIA on blood collected at necropsy (ad libitum fed state). Mean absolute concentrations ranged from 0.35 ± 0.04 to 0.59 ± 0.10 μg/L for insulin, and from 27.15 ± 2.65 to 76.53 ± 18.80 pg/mL for glucagon in F1. Corresponding values for F0 were 0.44 ± 0.07 to 0.50 ± 0.07 μg/L for insulin, and 50.76 ± 9.20 to 91.28 ± 23.17 pg/mL for glucagon. Plasma insulin levels were significantly lower in F1 exposed to 0.1 mg/kg DE-71 and trended lower in those exposed to 0.4 mg/kg mice relative to VEH/CON (Fig. 6a). Exposed F1 females also showed lower plasma glucagon at 0.4 mg/kg but showed no changes in GLP-1 relative to VEH/CON (Fig. 6b,c). In exposed F0, mean plasma insulin was also downregulated in 0.1 mg/kg dose group and mean plasma glucagon trended higher in 0.4 mg/kg dose only. F0 also showed significantly upregulated plasma GLP-1 at 0.1 mg/kg DE-71. Hence, the 0.1 mg/kg F1 group showed the most prominent diabetogenic phenotype and also the most downregulated levels of insulin. At 0.4 mg/kg, DE-71 produced a less pronounced glucose intolerance and an apparent reduction in insulin and reduced glucagon.

### Upregulated adrenal epinephrine content and reduced BAT after DE-71 exposure

**Figure 7:**
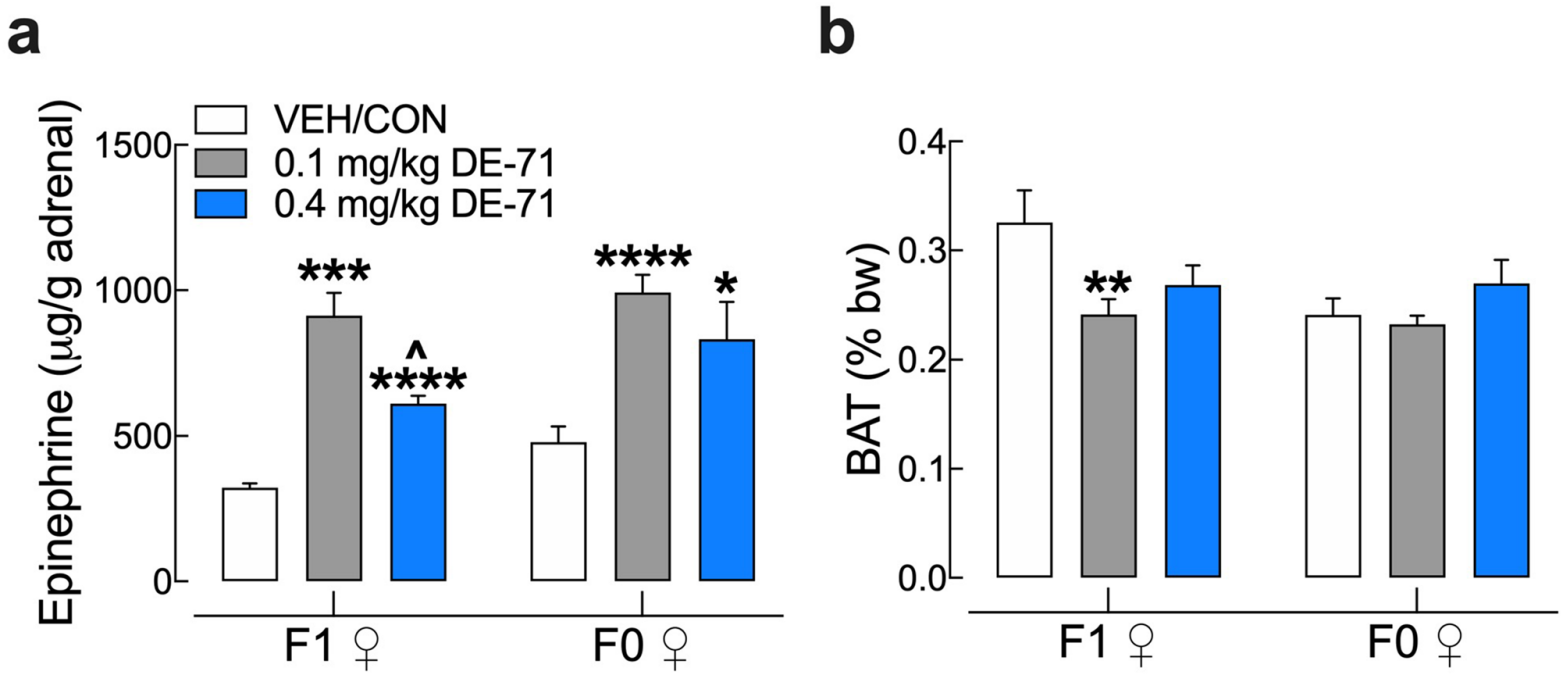
DE-71 exposure increases adrenal epinephrine content in F0 and F1 females and decreases brown adipose tissue (BAT) mass in F1 female mice. (**a**) Epinephrine content measured in adrenal glands harvested at necropsy was elevated in F0 and F1 females exposed to either dose of DE-71. No change in adrenal weight was measured (data not shown). (**b**) Interscapular brown adipose tissue (BAT) collected at necropsy was expressed as a percent of body weight. DE-71 exposure produces reduced BAT mass in F1 at 0.1 mg/kg. No effects were seen in F0. *Indicates significantly different from VEH/CON (*P < .05, **P < .01, ***P < .001, ****P < .0001); ^indicates significantly different from 0.1 mg/kg DE-71 (^*P* < 0.05). Dunnett’s T3 or Tukey’s post-hoc tests were used. Bars and error bars represent mean ± s.e.m. n = 4–8/group for epinephrine and n = 11–32/group for BAT. *BAT* brown adipose tissue, *F1* female offspring, *F0* dams.

Due to the important role of epinephrine in glucose and lipid homeostasis we examined if adrenal content was altered in glucose dysregulated mice exposed to DE-71. Control epinephrine levels were similar to those reported previously for adrenal gland in male mice^47^. Exposure to DE-71 significantly elevated adrenal epinephrine in both dams and female offspring, especially at 0.1 mg/kg dose (Fig. 7a). Adrenal weights were not different across experimental groups (data not shown). Brown adipose tissue (BAT) activity increases energy expenditure and has been inversely associated with diabetes and fasting glucose level^48^. When normalized to body weight, the mean intrascapular BAT mass was significantly decreased by 19% in 0.1 mg/kg exposed F1 relative to VEH/CON (Fig. 7b). There were no significant differences due to DE-71 exposure in F0.

### DE-71 exposure alters hepatic carbohydrate metabolic enzymatic activity

**Figure 8:**
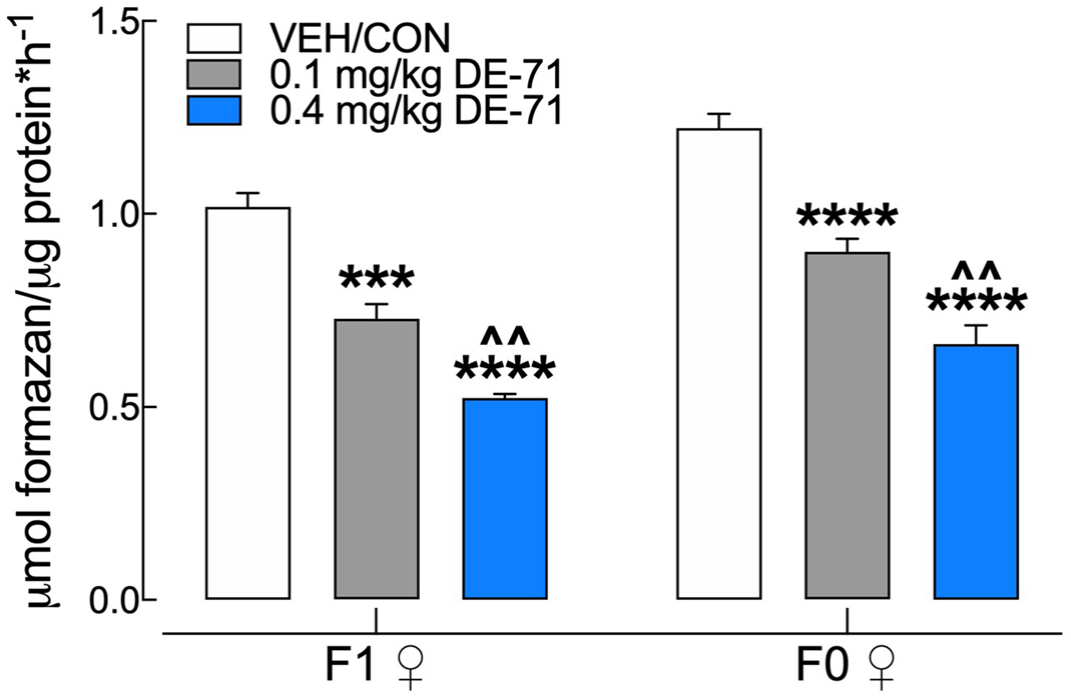
DE-71 exposure reduces hepatic activity of glutamate dehydrogenase (GDH) in F1 and F0 female mice. DE-71 exposure decreased GDH activity in F1 and F0 in a dose-dependent manner, producing greater reduction in the 0.4 mg/kg dose groups. *Indicates significantly different from VEH/CON (***P < .001, ****P < .0001); ^indicates significantly different from 0.1 mg/kg DE-71 (^^*P* < .01). Dunnett's T3 (F1) or Tukey’s (F0) post-hoc tests were used. Bars and error bars represent mean ± s.e.m. n = 6–8/group. *GDH* glutamate dehydrogenase, *F1* female offspring, *F0* dams.

Elevated glucose levels may be due to hepatic glucose production. Therefore, we tested the hypothesis that PBDEs increase the activity of glutamate dehydrogenase (GDH), a hepatic gluconeogenic enzyme important in normal glucose homeostasis. We found that exposure to DE-71 significantly *reduced* enzymatic activity of GDH in F0 and F1 (Fig. 8). DE-71 was significantly more effective at 0.4 mg/kg than at 0.1 mg/kg in both F1 and F0.

### DE-71 exposure increases hepatic levels of endocannabinoids (EC) and related fatty acid-ethanolamides in exposed F1 but not F0 female mice

**Figure 9:**
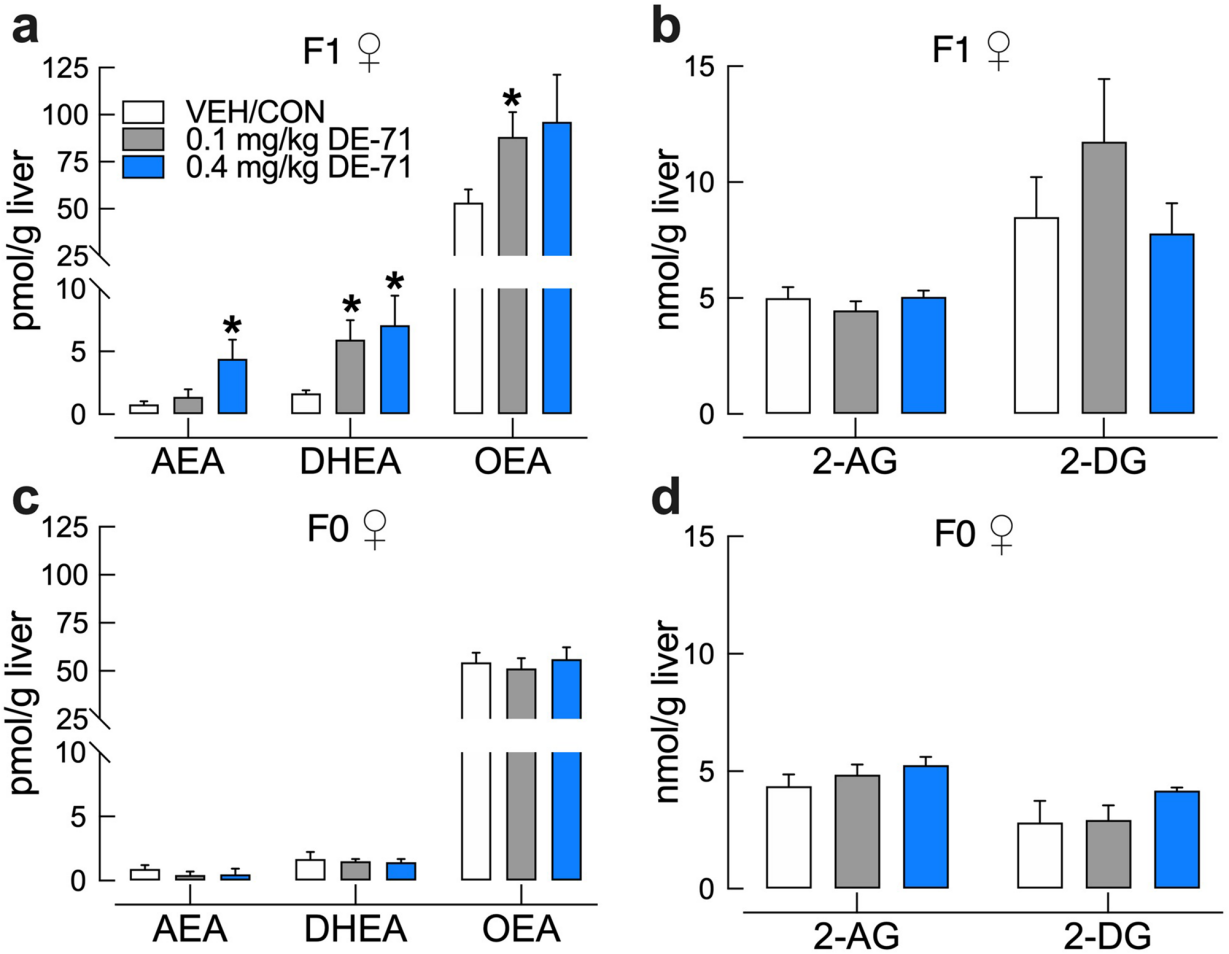
DE-71 exposure increases hepatic levels of endocannabinoid (EC) and related fatty acid-ethanolamides in exposed F1 but not F0 female mice. *Post mortem* liver tissue was analyzed using UPLC/MS/MS. Perinatal (**a**, **b**) but not adult exposure to DE-71 (**c**,**d**) produced elevated levels of hepatic ECs and fatty acid-ethanolamides. F1 exposed to 0.1 mg/kg displayed elevated DHEA and OEA (**a**). F1 exposed to 0.4 mg/kg showed elevated AEA (Anandamide), a primary EC, and DHEA. No significant differences were detected after adult exposure to DE-71 in F0 (**c**,**d**). The other primary EC, 2-AG and 2-DG did not exhibit changes in DE-71 exposed F1 (**b**) or F0 mice (**d**). *Indicate significantly different from VEH/CON (*P < .05). Bars and error bars represent mean ± s.e.m. n = 6–16/group for F1 and n = 3–5/group for F0. Dunnett’s T3 and Tukey’s *post-hoc* test was used. *AEA* arachidonoylethanolamide (Anandamide), *DHEA* docosahexanoyl ethanolamide, *OEA* n-oleoyl ethanolamide, *2-AG* 2-arachidonoyl-*sn*-glycerol, *2-DG* monoacylglycerol 2-docosahexaenoyl-*sn*-glycerol, *F1* female offspring, *F0* dams.

F1 exposed to either dose of DE-71 displayed altered liver levels of the endocannabinoid (EC), anandamide (arachidonoylethanolamide, AEA), and related fatty acid-ethanolamides, docosahexaenoyl ethanolamide (DHEA), and n-oleoylethanolamide (OEA), a shorter analogue of AEA, when compared to VEH/CON, with DHEA and OEA being more sensitive as indicated by sensitivity to the 0.1 mg/kg dose (Fig. 9a). In contrast, no changes were detected for the other primary EC, 2-arachidonoyl-sn-glycerol (2-AG), and related monoacylglycerol, 2-docosahexaenoyl-sn-glycerol (2-DG) (Fig. 9b). None of the lipids were altered by DE-71 in exposed F0 females as compared to VEH/CON (Fig. 9c,d).

## Discussion

The diabetogenic effects of PBDEs are not well understood. The objective of this study was two-fold. First, we explored whether the environmentally relevant industrial PBDE mixture DE-71, at chronic low doses, could influence in vivo and ex vivo biomarkers of diabetes. Secondly, we examined the diabetogenic potential of DE-71 exposure in dams and their female offspring. Our main finding is that developmental exposure to DE-71 produces significant glucose dysregulation including fasting hyperglycemia, impaired glucose tolerance, insulin insensitivity and delayed glucose clearance/utilization in response to insulin; all symptoms used clinically to diagnose diabetes and validate diabetic animal models^44^.

Our results show a more substantial glucose dyshomeostasis in exposed F1 than in F0 females. FBG was elevated in both 0.1 mg/kg (9 h fast) and 0.4 mg/kg F1 groups (11 h fast) compared to VEH/CON. In addition, F1 females exposed to 0.1 mg/kg showed glucose intolerance after a glucose challenge, such that glycemia reached a greater peak and recovery time to basal levels was extended. This abnormal phenotype was not observed in 0.4 mg/kg when expressing glucose levels as a percent of baseline. A similar but less prominent phenotype was observed in F0. The greater glycemia was unlikely due to enhanced hepatic glucose production from amino acids since DE-71 exposure caused significant reduction in the activity GDH, a key hepatic enzyme regulating amino acid-derived gluconeogenesis^49^. Similar downregulation by DE-71 has been reported for another gluconeogenic enzyme, phosphoenolpyruvate carboxykinase (PEPCK)^50^. Instead, the exaggerated peak glycemia after glucose challenge and the delay in the return to baseline blood glucose could have resulted from reduced plasma insulin, reduced insulin sensitivity and/or reduced lean mass (muscle, brain and liver), the principal site of glucose disposal^51^.

Another measure of abnormal glucose homeostasis found in 0.1 mg/kg exposed F1 was insulin insensitivity measured as a significant reduction in the blood glucose reduction rate (K_ITT_) compared to VEH/CON. F1 exposed to either DE-71 dose also showed an increased latency to reach minimum glycemia, which may indicate reduced glucose clearance/utilization. Finally, when expressing glucose levels as a percent of baseline, the glycemic response to insulin challenge showed a slower recovery at 60, 90 and 120 min post injection in F1 exposed to 0.1 and 0.4 mg/kg DE-71 relative to VEH/CON. In support of possible DE-71-induced insulin insensitivity, others have reported an increased glucose:insulin ratio in DE-71 exposed rats^50^. BDE-28 and penta-PBDE have been shown to reduce insulin signaling in adipocytes from insulin-resistant obese humans^36^ and rats^32^, respectively. Moreover, epigenetic/genetic changes in the liver of BDE-47-exposed rat offspring have been associated with insulin signaling and a canonical pathway related to Type 1 diabetes (T1D)^26,28^. Compared to the diabetogenic phenotype of F1 females, adult-exposed dams did not display fasting hyperglycemia nor an abnormal glycemia response to insulin and showed a more subtle glucose intolerance on IPGTT. These results suggest that developmental exposure to environmentally relevant PBDE congeners increases the risk of developing T2D later in life.

Previous experimental studies examining measures of glucose in developmentally exposed female rodents have reported results discordant with ours. No changes in measures of insulin or glucose action were found in female (or male) F1 exposed to a complex BFR mixture found in house dust: DE-71, DE-79, BDE 209 and hexabromocyclododecane (HBCDD) via the mother^52^ possibly explained by a net masking effect of individual congener actions. Using perinatal exposure to BDE-47, Suvorov and colleagues^53^ reported an improvement in glucose uptake on oral GTT in male but not female rat offspring, suggesting sex-specific effects. The differential results of these studies relative to ours may be explained by different perinatal dosing paradigms using dams, such as DE-71 via oral treats vs. BDE-47 via intravenous injection, respectively, and chronic doses of 0.1 and 0.4 mg/kg vs. 6 doses of 0.002 and 0.2 mg/kg bw, respectively. Another study performed in adult females reported no glucose intolerance in *virgin* female rats exposed to BDE-47 in adulthood^54^. In contrast, our exposed adult F0 showed slight but significant glucose intolerance during IPGTT suggesting that they may be more vulnerable to DE-71 under conditions of pregnancy and lactation. Alternatively, the relatively greater susceptibility of exposed F0 females in our study may be due to the combination of PBDE congeners present in DE-71. In adult male rats and mice BDE-47 treatment produced hyperglycemia in one study and diabetic symptoms in others but only when paired in two-hit models^30,31^. Interestingly, our results using DE-71 are similar to those produced by two brominated flame retardants not found in DE-71, BDE-209 (hyperglycemia with reduced insulin)^28^ and Firemaster-550 (apparent glucose intolerance)^27^.

Given the endocrine-disrupting properties of PBDEs^15,55,56^ we hypothesized that DE-71 would disrupt levels of glucoregulatory hormones that serve as diagnostic biomarkers for diabetes. Studies have shown that levels of insulin, glucagon and glucagon-like peptide 1 (GLP-1) are altered in T2D leading to pathological glucose dyshomeostasis^57^. Insulin and glucagon both normalize blood glucose levels under conditions of high and low plasma glucose, respectively. Insulin action facilitates cellular absorption of glucose from the blood whereas glucagon triggers the release of glucose into the blood from liver stores. Reduced plasma insulin in F1 after exposure to 0.1 mg/kg DE-71 may contribute, in part, to the pronounced glucose intolerance seen in this group. Epidemiological studies measuring insulin have reported conflicting reports, either no association with ∑PBDEs (BDE-47 and -153) in Canadian indigenous populations^33^ or a positive association between omental fat levels of BDE-28 and -99 and fasting insulin secretion in obese individuals^36^. Animal studies have failed to show changes in insulin levels with adult exposure to BDE-47^29^ or postnatal exposure to BDE-47^31^ or perinatal exposure to a mixture of BFRs, including PBDEs and HBCDD^52^. In 0.4 mg/kg F1 insulin reduction was less pronounced, and glucagon was decreased, which could explain the less marked glucose intolerance. This coupled with other changes, such as the lack of BAT mass reduction and/or the differential profile of hepatic endocannabinoids at this dose (see below) may help explain dose-related differences in glucose tolerance. In F0 exposed to 0.1 mg/kg DE-71 produced a significant increase in GLP-1 concomitant with glucose intolerance rather than an insulinotropic effect on glycemia. The inhibitory effects of DE-71 on both glucagon and insulin may indicate an altered capacity of α- and β-cell function in the pancreas, respectively. Interestingly, adult exposure to BDE-47 reduced gene expression for the rat GLP-1 receptor^29^. Glucoregulatory hormones were measured in an ad libitum fed state. Changes due to DE-71, especially for glucagon, may be even more exaggerated during fasting although this possibility must be examined in future studies.

Because of its important role in glucose metabolism and regulation of glucoregulatory hormones we examined if adrenal epinephrine was impacted by DE-71 exposure and found elevated content as compared to VEH/CON especially at 0.1 mg/kg. In F1, this dose also produced fasting hyperglycemia, glucose intolerance, insulin insensitivity and low plasma insulin in exposed F1 (Fig. 4), suggesting the possibility that DE-71 actions on glucose intolerance and insulin reduction and upregulation of the sympathoadrenal system may be related since diabetic animals show elevated adrenal epinephrine produced in chromaffin cells^58^. For example, epinephrine stimulates gluconeogenesis in liver, stimulates glycogenolysis either directly or by facilitating glucagon action and inhibits insulin^59^. Interestingly, penta-BDEs enhance adrenergic-stimulated actions in rat adipocytes^32^. Previous reports of in vitro exposure to PBDE (and PCBs) showed opposite changes in catecholamine (CA) levels in and/or release from cultured chromaffin cells depending on the congener^60,61^. A limitation of our study is that we did not measure plasma levels of epinephrine, although it seems feasible that adrenal and plasma epinephrine may be co-regulated^62^. However, DE-71 may have direct effects on epinephrine content in the adrenal gland without consequences on plasma epinephrine levels. Perinatal DE-71 exposure appears to exaggerate adrenal mRNA levels of the major catecholamine synthetic enzyme, tyrosine hydroxylase, in rats (unpublished observations, Spurgin and Currás-Collazo).

We also measured BAT, which is under the trophic influence of ß-adrenergic sympathetic- and insulin-mediated regulation and epinephrine^63–65^ Reduced intrascapular BAT mass was uniquely found in 0.1 mg/kg DE-71-exposed F1 as compared to VEH/CON. Lower BAT mass may contribute to fasting hyperglycemia and reduced glucose clearance^48^ and is negatively associated with central obesity and diabetes^63,64,66^. In correspondence, BAT activation of lipolysis and thermogenesis protects against these^67^. Further studies are needed to determine how BAT participates in metabolic health and PBDE-induced glucose dyshomeostasis.

Unbalanced energy homeostasis, including hyperglycemia caused by either diet-induced obesity or T2D diabetes, is associated with elevated concentrations of endocannabinoids (ECs) in liver, visceral fat, serum, pancreas and small intestine epithelium^68–70^. These endogenous lipid molecules act via CB_1_Rs in liver to induce glucose production by increasing gluconeogenic genes and promoting fatty acid synthesis^69,71^. Hepatic CB_1_Rs also participate in insulin signaling and glucose uptake^72^. Pharmacological blockade of CB_1_Rs significantly reduces hyperglycemia, improves glucose tolerance and/or insulin sensitivity in obese diabetic Zucker rats or diet-induced obese mice and humans^73–75^. In support of a role of ECs in a T2D diabetogenic phenotype, our data demonstrate that mice with the most glucose dysregulation (DE-71-exposed F1) display unique increases in levels of the EC, AEA, and related fatty acid-ethanolamides, DHEA and OEA, in liver. F1 exposed to 0.1 mg/kg DE-71 showed increased DHEA and OEA but not in AEA. While it is unclear why this group did not show increased AEA levels relative to VEH/CON, it did show increased DHEA, which is likely an agonist for CB1 and CB2 receptors, indicating that the EC system may participate in the diabetogenic phenotype. The upregulated levels of OEA, which is also an agonist at PPARα receptor pathway, may provide protective effects^76^. Future studies using select antagonists of CB1, CB2, or PPARα receptors could delineate the role of these ECs in DE-71 diabetogenic phenotype. In contrast to AEA, levels of the other primary EC, 2-arachidonoylglycerol (2-AG), in the liver were not different among groups. Indeed, levels of AEA and 2-AG are not always equally impacted by experimental interventions, which may result from differential regulation of individual EC metabolic pathways^77–79^. It should be noted that similar EC system changes in pancreas (not measured here) may contribute to the relative deficiency of plasma insulin that we report for DE-71 exposed F1 females^80^.

Congener profiles in the liver were of particular interest since the liver is a key organ regulating glucose homeostasis and xenobiotic metabolism. Mean values for ∑PBDEs concentration were ~ 15-fold lower in exposed F1 (~ 200 ng/g lw) than in exposed F0 (~ 2900 ng/g lw) at the 0.1 mg/kg/d dose. Liver levels in exposed F1 are in the range of maximum values reported for human ∑PBDEs concentration in serum (typically lower than liver) of North American populations including Canadian indigenous Inuits and Crees (max 219–402 ng/g lipid wt) and California U.S. women (max ~ 749.7 ng/g lipid wt)^12,33^. However, they are 5- to 10-fold greater than those in human breast milk and serum measured in different parts of the world in recent years (in ng/g lipid wt): UK 2014 (15)^81^ and China 2016–2017 (2.87)^34^ and California 2009–2012 (41.5)^82^.

The composition of 9 congeners previously detected in the DE-71 lot used has been reported: BDE-99 (45.3%), -47 (33.3%), -100 (8.2%), -153 (3.6%), -154 (3.2%), -85 (2.5%), -139 (0.9%), -138 (0.5%), -28 (0.2%), and -17 (0.1%)^17^. We also detected BDE-66 and -183 but did not detect -138, all of which have been reported in trace amounts in previous studies^41^. In our study, BDE-99 and -153 were the dominant penetrant congeners followed by BDE-47 and -100 in 0.1 mg/kg exposed F0. These were the same primary congeners reported in the DE-71 lot used in this study. Similar results on predominant congeners (BDE-47, -99, -100 and -153) have been found in women sampled recently^12,34,36,81,83^. The fast elimination of BDE-47 from livers of mice^43,84^ and debromination to lower-brominated congeners such as BDE-28 may explain the lower content of BDE-47 in dam liver relative to that of BDE-99 and -153. In contrast to F0, livers of exposed F1 displayed a smaller set of congeners, i.e., only BDE-28/33 and BDE-153. To our knowledge, no previous studies have determined the complete congener profile in female offspring liver after exclusive indirect exposure to DE-71 via maternal transfer. The observation of disproportionately elevated levels of BDE-153 in F1 and F0 was not unexpected since, unlike BDE-47 and BDE-99, it lacks unsubstituted carbons resulting in poor metabolism by the body and allowing for quick absorption and tissue retention, especially in liver^43,85^.

The lack of hepatic BDE-47 and -99 in F1, may be due to the ultra-low doses used and and/or shorter exposure period that their mothers and/or elimination toxicokinetics in F0 dams receiving direct exposure^43,85^. However, transfer of BDE-47 from dams to offspring does occur during both gestation and lactation^86^ and disposed of more slowly in postnatal mouse pups^84^. One possible limitation of our study is that we measured offspring BDE levels in adulthood (~ 90 d after weaning), and by this time BDE-47 and other DE-71 congeners could have been eliminated, or hydroxylated and, therefore, not detected. Therefore, it is possible that the concentration and range of PBDE congeners were higher/different at a critical developmental period and at the time of the metabolic measurements. Also, we cannot rule out the possibility that impurities in DE-71 arising during production, such as polybrominated biphenyl (PBB) and polybrominated dibenzofurans (PBDFs)^87^, may contribute to diabetogenic effects reported, although evidence for the tetra-PBBs found in DE-71 is lacking.

It is likely that congeners found in F1 liver, BDE-153 and BDE-28, contribute significantly to the pronounced diabetic phenotype seen in DE-71-exposed F1. Adverse diabetogenic symptoms have been associated with both of these PBDE congeners in human serum, breastmilk and children^34–36^. BDE-153 has been positively associated with diabetes and/or MetS in studies of men and women in China^29,34^, in US^35^ and with fasting hyperglycemia in Canada^33^ but not in other populations^88–90^. In particular, Lim and colleagues^35^ reported that the positive association showed an inverted U-shape indicating a significant effect only at low and moderate PBDE exposure, which supports the dose-dependent hormesis seen in our study. Moreover, BDE-28 may contribute to insulin-resistant diabetes typical of T2D^36^. Gestational diabetes mellitus (GDM) in healthy US pregnant women sampled from 2013–2015 was also positively associated with high body burdens of BDE-153 and BDE-28^37,38^. Diabetes has increased to pandemic proportions worldwide during the last few decades and we speculate that PBDEs may act as MDCs contributing to this. Our findings may help inform about the potential risks of POP exposure during development contributing to the etiology of diabetes in adulthood.

In conclusion, we demonstrate that chronic, low-dose perinatal exposure to an environmentally relevant anthropogenic PBDE mixture, DE-71, produces multi-symptom effects related to diabetes: fasting hyperglycemia, glucose intolerance, abnormal sensitivity and glucose clearance after insulin challenge, and increased hepatic endocannabinoid tone, especially after perinatal exposure. DE-71 effects on F0 were more limited indicating that indirect exposure to developing offspring is more detrimental. Other glycemic control effects that may aggravate/accompany DE-71′s diabetogenic-promoting effects occur more generally in exposed F0 and F1, such as reduced insulin and altered glucoregulatory endocrines, exaggerated sympathoadrenal activity and reduced hepatic GDH enzymatic activity. These adverse health effects appear to be associated with maternal transfer of BDE-28 and BDE-153 to F1. Our results indicate that exposed F1 female mice are susceptible to metabolic reprogramming by DE-71 that leads to a diabetogenic phenotype persisting beyond the period of exposure. Our findings warrant additional animal studies that further characterize PBDE-induced diabetic pathophysiology and identify critical developmental windows of greater susceptibility. They should also inform human studies focused on assessing the risk of emerging adult metabolic disease associated with early life PBDE exposure especially in North American populations.

## Methods

### Animals

C57Bl/6N mice were generated using breeders obtained from Charles River (Raleigh, NC) or Taconic Biosciences (Germantown, NY). Mice were group housed 2–4 per cage and maintained in a non-specific pathogen free vivarium on a 12 h light/dark cycle at an ambient temperature (20.6–23.9 °C) and relative humidity environment (20–70%). Mice were provided rodent chow and municipal tap water ad libitum. Procedures on the care and treatment of animals were performed in compliance with the National Institutes of Health *Guide for the Care and Use of Laboratory Animals* and approved by the University of California, Riverside Institutional Animal Care and Use Committee (AUP#20170026).

### Dosing solutions

Technical pentabromodiphenyl ether (DE-71; Lot no. 1550OI18A; CAS 32534-81-9), a commercial mixture of PBDEs was originally obtained from Great Lakes Chemical Corporation (West Lafayette, IN). DE-71 dosing solutions were prepared to yield two ultra-low doses: 0.1 and 0.4 mg/kg bw per day. These doses contain a similar molar equivalent of BDE-47 used in previous mouse metabolic and neurobehavioral studies^30,91^. Dosing solution (0.05 g/L) was prepared from a stock solution (0.2 g/L) consisting of DE-71 dissolved in corn oil (Mazola) at 65 °C followed by sonication. Vehicle control solution (VEH/CON) contained corn oil without the addition of DE-71. Solutions were made fresh every 6 months.

### DE-71 exposure and animal procedures

Female virgin mice (PND 30–60) were introduced to cornflakes daily for 1 week. Dams were randomly assigned to one of three exposure groups: corn oil vehicle control (VEH/CON), 0.1 or 0.4 mg/kg bw per day DE-71 (Fig. 1). A 10-week dosing regimen was used that included ~ 4 weeks of pre-conception, plus gestation (3 weeks) and lactation (3 weeks). Offspring were weaned after the lactation period at PND 21 and housed in same-sex groups. This exposure paradigm was chosen to model human-relevant chronic, low-level exposure^34,81,92^. PBDE transfer from mother to infant has been shown to occur during gestation and lactation in humans^22^ and in rodent models^16,86^. Under this regimen, each dam received a daily exposure to DE-71 for an average of 70–80 d and offspring were exposed perinatally for 39 d via mother’s blood and milk. Dams were fed oral treats, (Kellogg’s Corn Flakes) infused with dosing solution (2 μL/g bw) daily, except on PND 0 and 1, a method established to ensure ingestion without the stress of oral gavage^86,93^. Consumption was visually confirmed and offspring co-housed with dams were never observed to ingest cornflakes. During the last week of the 4-week pre-conception exposure period dams were mated with an untreated C57Bl/6 N male. A 10-week dosing regimen was used as described^86^ to ensure maternal bioaccumulation prior to conception, especially a concern at low doses. In a subset of dams, gestational weight gain and food intake was monitored daily from GD15-18. F0 and F1 female offspring were used in vivo and ex vivo for analysis of physiological, metabolic and endocrine parameters (Fig. 1). Metabolic endpoints for F0 were chosen to be 1–2 weeks post-lactation, at which time dams were ~ 5 months of age. In order to compare the adult phenotype of F1 these were tested at a comparable age of 4 months. During sacrifice, under terminal isoflurane anesthesia, cardiac blood (0.3–1 mL) was collected and animals were cervically dislocated. Plasma was centrifuged at 16,000 × *g* for 20 min at 4 °C. A cocktail of protease inhibitors and EDTA was added to the plasma fraction and samples stored at − 80 °C until immunoassay analyses. Liver, pancreas, spleen, adrenal glands and interscapular BAT were excised and weighed. Plasma, liver and adrenal samples were snap-frozen over dry ice and stored at − 80 °C for later analysis of PBDE congener tissue level determination, plasma endocrines, adrenal epinephrine, liver endocannabinoids and enzymatic activity.

### Congener analysis via mass spectrometry

The concentration of 29 BDE congeners in liver samples collected at sacrifice were determined using gas chromatography/mass spectrometry operated in electron capture negative ionization mode (ECNI) as previously described^94^. Samples were analyzed for nine congeners that comprise the DE-71 lot used as described^17^ (BDE-17, 28/33, -47, -85/155, -99, -100, -138, 153, -154) as well as -25, -30, -49, -66, -71, -75, -116, -119, -156, -181, -183, -190, -191, -200/203, -205, -206, -209 that are commonly monitored in the environment. There are only 6 congeners that make up the great majority (96%) of the DE-71 mixture used here: BDE-99 > -47 > -100 > -153 > -154 > -85 that range from 45.0 to 2.54% w/w^17^. Similarly, 5 of these are the most prevalent BDE congeners found in human tissues: BDE-28, -47, -99, -100, and -153^95^ (Center for Disease Control and Prevention, NHANES, 2015). Prior to extraction, two internal standards were spiked into the homogenized tissue, including a monofluorinated BDE congener (F-BDE-69; Accustandard Inc.) and isotopically labeled BDE-209 (^13^C BDE-209; Wellington Laboratories). BDE congeners were extracted from liver tissues (approximately 1 g) using sonication and an aliquot of the extract was used for gravimetric analysis of lipid content. The remaining extract was purified using a Florisil solid phase extraction cartridge. Extracts were spiked with a third standard, isotopically labeled chlorinated diphenyl ether (^13^C CDE-141; Wellington Laboratories) to measure recovery of the internal standards and then analyzed via gas chromatography mass/ spectrometry using ECNI with methane as a reagent gas as previously described. BDE concentrations are expressed as ng/g lipid weight. The method detection limit (MDL) was calculated using 3 times the standard deviation of the laboratory blanks and was equivalent to 0.5 ng/g lw. Recoveries of F-BDE-69 averaged 90.9% ± 6.9% while ^13^C BDE 209 averaged 106% ± 19%.

### Glucose tolerance test (IPGTT) and insulin tolerance test (ITT)

To perform the intraperitoneal (ip) glucose tolerance test (IPGTT), mice were fasted overnight (ON) for 11 h during the metabolically active dark cycle and then injected with glucose (2.0 g/kg bw, i.p.). Tail blood (1 μL) was collected and glucose sampled at time 0 (FBG) and at 15, 30, 60, and 120 min post-glucose challenge. Plasma glucose concentrations were measured using a calibrated glucometer (OneTouch Ultra 2, LifeScan Inc.). Seven days following IPGTT, an insulin tolerance test (ITT) was performed on mice fasted ON for 9 h and then injected with Humulin R bolus (0.5 U/kg bw, i.p.). Tail blood was collected and glucose sampled at time 0 (FBG), 15, 30, 45, 60, 90 and 120 min post-injection. The area under (AUC) or above the glycemia curve (inverse AUC) was calculated over 0–120 min post injection. To determine in vivo insulin sensitivity, the blood glucose reduction rate after insulin administration, K_ITT_, was calculated using the formula 0.693 × t_1/2_^–1^. Half-life (t_1/2_) was calculated from the slope of the blood glucose concentration from 0–15 min post insulin injection, when plasma glucose concentration declines linearly^51^.

### Immunoassays

Plasma collected via cardiac puncture at each necropsy (ad libitum fed state) was analyzed for several peptides using commercially available kits according to manufacturer’s instructions. Plasma insulin was measured using commercial ELISA kits (ALPCO and Mercodia). Colorimetric reaction product was read as optical density at 450 nm on a plate reader. The Mercodia assays had a sensitivity of 0.15 mU/L and 1 mU/L in a dynamic range of 0.15–20 mU/L and 3–200 mU/L. The inter-assay coefficient of variability (CV) was 4.9 and 3.4% and intra-assay CV was 5.1 and 4.5%, respectively. The ALPCO insulin ELISA had a sensitivity of 0.019 ng/mL in a dynamic range of 0.025–1.25 ng/mL and inter- and intra-assay CV of 5.7% and 4.5%, respectively. Active glucagon-like peptide-1 (7–36) Amide (GLP-1) was detected by indirect sandwich amide chemiluminescence ELISA (ALPCO) using a luminescence plate reader. This assay had an analytical sensitivity of 0.15 pM in a dynamic range of 0.45–152 pM and inter- and intra-assay CV of 11.6 and 9.5%, respectively. Glucagon was measured by chemiluminescence ELISA (ALPCO), which has a similar assay principle as the GLP-1 assay. This assay has a sensitivity of 41 pg/mL in a dynamic range of 41–10,000 pg/mL and inter- and intra-assay CV of 9.8% and 7.6%, respectively. All kits were specific to rat/mouse hormones. Plasma insulin, active GLP-1 and glucagon were determined by interpolating absorbance or luminosity values using a 4-parameter-logarithmic standard curve.

### Ultra-performance liquid chromatography-tandem mass spectrometry

Hepatic lipids were extracted following a modification of the Folch method^96^. In brief, samples of flash-frozen liver tissue were weighed (10–20 mg) and homogenized in 1 mL of methanol solution containing 500 pmol d_5_-2-arachidonoyl-*sn*-glycerol, 10 pmol d_4_-oleoyloethanolamide, and 1 pmol d_4_-arachidonoylethanolamide as internal standards followed by the addition of 2 mL of chloroform, 1 mL of water, and centrifuged at 2000 × *g* for 15 min at 4 °C. The organic phase was removed and subjected to chloroform extraction. The pooled lower phases were dried under N_2_ gas and resuspended in 0.1 mL methanol:chloroform (9:1). A 1 μL injection was used for analysis of the EC, arachidonoylethanolamide (AEA) and 2-arachidonoyl-sn-glycerol (2-AG), and related fatty acid ethanolamides, docosahexaenoyl ethanolamide (DHEA) and n-oleoylethanolamide (OEA), and monoacylglycerol, 2-docosahexaenoyl-sn-glycerol (2-DG), respectively, was performed via ultra-performance liquid chromatography coupled to tandem mass spectrometry (UPLC/MS/MS) as previously described by us^97^.

### Glutamate dehydrogenase (GDH) activity

GDH is a key enzyme bridging amino acid-to-glucose pathways. GDH activity in crude liver homogenates was assayed using the tetrazolium salt method of Lee and Lardy^98^ with modification for multi-well plates^99^. The 5% (w/v) homogenates of liver (5 mg) were prepared in 0.25 M sucrose solution. The reaction mixture contained 50 μmol/l of substrate (sodium glutamate), 100 μmol/l of phosphate buffer (pH 7.4), 2 μmol/l of iodonitrotetrazolium chloride and 0.1 μmol/l of NAD and distilled water. The reaction, in all the samples, was started by the addition of sample liver homogenate and proceeded for 30 min at 37 °C. The formazan formed was measured as optical density at 545 nm then converted to concentration using a iodonitrotetrazolium formazan (TCI) standard curve fitted with a linear regression model^99^. Protein content was measured using a bicinchoninic acid assay (ThermoFisher). The activity of GDH was expressed as µmol formazan formed per ug protein per h. Samples were run in duplicate.

### Epinephrine assay

Epinephrine content in adrenal glands was measured using a modification of the trihydroxyindole method. Using this method of catecholamine oxidation at 0 °C, Kelner and colleagues^100^ obtained values for epinephrine content in bovine chromaffin cell lysates that were nearly identical to those measured using HPLC/electrochemical determination. Briefly, 5 mg of adrenal tissue homogenates (in 200 µL of 0.05 N perchloric acid) were centrifuged at 15,000 × *g* at 0 °C for 15 min. Sample supernatant (30 μL) was added to 10% acetic acid (pH 2). Then 60 µL of 0.25% K_2_Fe(CN)_6_ was added to each sample and the mixture was incubated at 0 °C for 20 min. The oxidation reaction was stopped by the addition of 60 µL of a 9 N NaOH solution containing 4 mg/mL ascorbic acid (alkaline ascorbate). Fluorescence emission was determined at 520 nm (excitation wavelength at 420 nm) using a fluorescence plate reader. Each sample yielded mean fluorescence intensity units that were converted into epinephrine concentration expressed as μg/g adrenal wet weight using calibration standards and polynomial curve fitting.

### Statistical analysis

Data are presented as mean ± s.e.m. An unpaired, two-tailed Student’s t-test and Mann–Whitney U test were used for two group comparisons of K_ITT_ and ∑PBDEs congener data, respectively. A one-way analysis of variance (ANOVA) was used to test the main effect of one factor in more than two groups. A Brown-Forsythe ANOVA was used instead if the group variances were significantly different. When normality assumption failed (Shapiro–Wilk test) a non-parametric test was used (Kruskal–Wallis H test). Data for fasting glycemia were analyzed by two-way ANOVA. ITT and IPGTT experiments were analyzed by repeated measures two-way or mixed model ANOVA. The Geisser-Greenhouse correction was used in some cases as noted. ANOVA was followed by post hoc testing for multiple group comparisons while reporting multiplicity-adjusted *P* values. Statistical analyses were performed using GraphPad Prism v.8.4.3. Differences were considered significant at *P* < 0.05. Additional statistical results can be found in Supplementary Statistical Results online.

### Disclaimer

J.M.K. is now a 2nd Lieutenant at the Uniformed Services University, Department of Defense. Her work was performed at the University of California, Riverside before becoming a military officer. However, we want to emphasize that the opinions and assertions expressed herein are those of the authors and do not necessarily reflect the official policy or position of the Uniformed Services University or the Department of Defense.

## Supplementary information

Supplementary Information
