# Supplementary material for: Maternal transfer of environmentally relevant polybrominated diphenyl ethers (PBDEs) produces a diabetic phenotype and disrupts glucoregulatory hormones and hepatic endocannabinoids in adult mouse female offspring
Source: Sci Rep. 2020 Oct 22;10:18102. doi: 10.1038/s41598-020-74853-9 (PMC7582149; doi:10.1038/s41598-020-74853-9)
Supplement: Supplementary file 1 — Supplementary Information [file 41598_2020_74853_MOESM1_ESM.pdf]

Supplementary Information

Maternal Transfer of Environmentally Relevant Polybrominated Diphenyl Ethers (PBDEs) Produces a Diabetic Phenotype and Disrupts Glucoregulatory Hormones and Hepatic Endocannabinoids in Adult Mouse Female Offspring

Elena V. Kozlova[1], Bhuvaneswari D. Chinthirla[1], Pedro A. Pérez[2], Nicholas V. DiPatrizio[2], Donovan A. Argueta[2], Allison L. Phillips[3], Heather M. Stapleton[3], Gwendolyn M. González[1], Julia M. Krum[1], Valeria Carrillo[1], Anthony E. Bishay[1], Karthik R. Basappa[1] and Margarita C. Currás-Collazo[1,*]

[1]Department of Molecular, Cell and Systems Biology, University of California Riverside, Riverside, California USA
[2] Division of Biomedical Sciences, School of Medicine, University of California Riverside, Riverside, California, USA
[3] Duke University, Nicholas School of the Environment, Durham, North Carolina, USA

***Corresponding author**:
Dr. Margarita C. Currás-Collazo, Ph.D., FAPS
Associate Professor of Neuroscience
Department Molecular, Cell and Systems Biology
University of California, Riverside
Riverside, CA 92521
951-827-3960
mcur@ucr.edu

Supplementary Table S1. Dam Gestational Parameters and Litter Outcomes After Chronic Low Dose exposure to DE-71

	**VEH/CON**	**0.1 mg/kg/d DE-71**	**0.4 mg/kg/d DE-71**
**Maternal Parameters**			
n[a]	7	8	11
Gestational food intake<br>Absolute (g/day)<br>Relative (g/pup)	 6.83 ± 0.73<br>1.20 ± 0.20	 6.30 ± 0.34<br>1.35 ± 0.32	 6.67 ± 0.31<br>1.05 ± 0.15
Gestational weight gain<br>Absolute (g)<br>Relative (g/pup)<br>Relative to initial (%)[b]	 13.98 ± 1.29<br>2.39 ± 0.27<br>70.50 ± 7.03	 13.76 ± 0.57<br>2.84 ± 0.55<br>66.48 ± 4.10	 13.70 ± 1.23<br>2.15 ± 0.37<br>65.40 ± 5.46
**Litter Parameters**			
n	16	19	18
Litter size	5.81 ± 0.57	5.79 ± 0.32	7.17 ± 0.51
Females/litter	2.56 ± 0.29	3.26 ± 0.33	3.61 ± 0.29
Males/litter	3.25 ± 0.48	2.68 ± 0.32	3.56 ± 0.40
Secondary sex ratio (M/F)	0.54 ± 0.04	0.46 ± 0.05	0.48 ± 0.04

[a]indicates number of dams per treatment group
[b]Initial weight was taken at 18 days before pup birth
Data are expressed as mean±s.e.m. Food intake was measured from GD13-GD18. Maternal weight gain was calculated as difference in weight from pre-pregnancy to late gestation (GD 16-18). Relative weight gain was normalized to pre-pregnancy weight (initial) and to total pups in litter (relative). Litter size was measured at PND0 to avoid the confound of infanticide typical of primiparous C57BL/6 dams. The secondary sex ratio was calculated as the proportion of the total pups that are male.

Supplementary Table S2. Mass Spectrometric Analysis (GC/ECNIMS) of PBDE congeners in DE-71-exposed mouse dams and their female offspring

Compound/Substituents	IUPAC Number	Dams			Female Offspring		
		VEH/CON	0.1 mg/kg DE-71	0.4 mg/kg DE-71	VEH/CON	0.1 mg/kg DE-71	0.4 mg/kg DE-71
n		4	4	3	4	4	4
2,2′,4-tri BDE	BDE 17	<MDL	<MDL	<MDL	<MDL	<MDL	<MDL
2,3’,4-tri BDE	BDE 25	<MDL	<MDL	<MDL	<MDL	<MDL	<MDL
2,4,4′-tri BDE, 2′,3,4-tri BDE	BDE 28, 33	<MDL	16.9 ± 4.05*	13.0 ± 2.55[b]	<MDL	7.04 ± 3.93	22.8 ± 8.15*
2,4,6,-tri BDE	BDE 30	<MDL	<MDL	<MDL	<MDL	<MDL	<MDL
2,2′,4,4′-tetra BDE	BDE 47	<MDL	422 ± 110*	192 ± 151	<MDL	<MDL	<MDL
2,2′,4,5′-tetra BDE	BDE 49	<MDL	<MDL	<MDL	<MDL	<MDL	<MDL
2,3′,4,4′-tetra BDE	BDE 66	<MDL	17.9 ± 6.18	14.6 ± 8.05	<MDL	<MDL	<MDL
2,3′,4′,6-tetra BDE	BDE 71	<MDL	<MDL	<MDL	<MDL	<MDL	<MDL
2,4,4′,6-tetra BDE	BDE 75	<MDL	<MDL	<MDL	<MDL	<MDL	<MDL
2,2′,3,4,4′-penta BDE, 2,2′,4,4′,6,6′-hexa BDE	BDE 85, 155	<MDL	119 ± 11.0**	138 ± 31.2***	<MDL	<MDL	<MDL
2,2′,4,4′,5-penta BDE	BDE 99	<MDL	1305 ± 153**	743 ± 425	<MDL	<MDL	<MDL
2,2′,4,4′,6-penta BDE	BDE 100	<MDL	426 ± 41.7***	267 ± 86.9*	<MDL	<MDL	<MDL
2,3,4,5,6-penta BDE	BDE 116	<MDL	<MDL	<MDL	<MDL	<MDL	<MDL
2,3’,4,4’,6 –penta BDE	BDE 119	<MDL	<MDL	<MDL	<MDL	<MDL	<MDL
2,2′,3,4,4′,5′-hexa BDE	BDE 138	<MDL	<MDL	<MDL	<MDL	<MDL	<MDL
2,2′,4,4′,5,5′-hexa BDE	BDE 153	<MDL	652 ± 52.9**	1655 ± 206****,^^^	<MDL	205 ± 27.7*	1008 ± 102**,^
2,2′,4,4′,5,6′-hexa BDE	BDE 154	<MDL	57.4 ± 5.61*	68.9 ± 25.6*	<MDL	<MDL	<MDL
2, 3,3′,4,4′,5-hexa BDE	BDE 156	<MDL	<MDL	<MDL	<MDL	<MDL	<MDL
2,2′, 3,4,4′,5,6-hepta BDE	BDE 181	<MDL	<MDL	<MDL	<MDL	<MDL	<MDL
2,2′, 3, 4,4′,5′,6-hepta BDE	BDE 183	<MDL	39.6 ± 6.94**	29.5 ± 8.76*	<MDL	<MDL	<MDL
2,3,3′,4,4′,5,6-hepta BDE	BDE 190	<MDL	<MDL	<MDL	<MDL	<MDL	<MDL
2, 3,3′,4,4′,5′,6-hepta BDE	BDE 191	<MDL	<MDL	<MDL	<MDL	<MDL	<MDL
2,2’,3,3’,4,5,6,6’-octa BDE, 2,2′,3,4,4′,5,5′,6-octa BDE	BDE 200, 203	<MDL	<MDL	<MDL	<MDL	<MDL	<MDL
2, 3,3′,4,4′,5,5′,6-octa BDE	BDE 205	<MDL	<MDL	<MDL	<MDL	<MDL	<MDL
2,2′,3,3′,4,4′,5,5′,6-nona BDE	BDE 206	<MDL	<MDL	<MDL	<MDL	<MDL	<MDL
2,2′,3,3′,4,4′,5,5′,6,6′-deca BDE	BDE 209	<MDL	<MDL	<MDL	<MDL	<MDL	<MDL
ΣPBDEs		<MDL	206 ± 1.30	1017 ± 0.37	<MDL	2996 ± 1.26	2915 ± 1.57

Content was measured as ng/g lipid

Values in table are expressed as mean±s.e.m.

ΣPBDEs are reported are geometric mean ± geometric standard deviation

* Indicates a statistically significant n apparent difference relative to VEH/CON by Dunnet’s T3 *post hoc* test following Brown-Forsythe ANOVA or Tukey’s *post hoc* test following One-way ANOVA (*P*<.05)

^^^ Indicates significantly different relative to 0.1mg/kg group by Tukey’s *post hoc* test following One-way ANOVA (*P*<.001)

[a] vs VEH/CON 0.4mg/kg P=.06

BDE, brominated diphenyl ether; IUPAC, International Union of Pure and Applied Chemistry; MDL,method detection limit

Supplementary Table S3. Chronic Low Dose DE-71 Exposure Has Minimal Effects on Body and Selected Organ Weights and lowers BAT Weight

	**VEH/CON**	**0.1 mg/kg/d DE-71**	**0.4 mg/kg/d DE-71**
**Dams**			
n	23	26	21
Necropsy body weight	24.19 ± 0.36	24.02 ± 0.35	25.05 ± 0.59
Liver			
Absolute	1.10 ± 0.03	1.20 ± 0.04	1.11 ± 0.03
Relative	45.41 ± 0.89	49.75 ± 1.46*	44.33 ± 0.96^
Pancreas			
Absolute	0.17 ± 0.01	0.18 ± 0.008	0.17 ± 0.01
Relative	6.97 ± 0.34	7.60 ± 0.26	6.99 ± 0.39
Spleen			
Absolute	0.09 ± 0.004	0.09 ± 0.002	0.09 ± 0.004
Relative	3.86 ± 0.13	3.72 ± 0.11	3.74 ± 0.18
BAT (n=8-11)			
Absolute	0.06 ± 0.004	0.06 ± 0.004	0.07 ± 0.007
Relative	2.41 ± 0.15	2.32 ± 0.08	2.70 ± 0.22
**Female Offspring**			
n	22	30	28
Necropsy body weight	20.80 ± 0.44	19.42 ± 0.37*	21.73 ± 0.39^^^
Liver			
Absolute	0.90 ± 0.02	0.86 ± 0.02	0.99 ± 0.02*,^^^
Relative	43.41 ± 0.86	44.16 ± 0.75	45.55 ± 0.73
Pancreas			
Absolute	0.14 ± 0.005	0.14 ± 0.006	0.16 ± 0.006
Relative	6.95 ± 0.23	7.32 ± 0.25	7.30 ± 0.31
Spleen			
Absolute	0.08 ± 0.002	0.07 ± 0.003	0.07 ± 0.002
Relative	3.73 ± 0.11	3.75 ± 0.13	3.41 ± 0.10
BAT (n=13-33)			
Absolute	0.062 ± 0.006	0.049 ± 0.003*	0.054 ± 0.005
Relative	3.51 ± 0.38	2.41 ± 0.14*	2.68 ± 0.18

Body weights and organ weights and BAT (absolute weights) are in grams; organ/BAT-weight-to-body-weight ratios (relative weights) are given as mg organ weight/g body weight. Values are reported as mean±s.e.m.
^^^ Indicates significantly different relative to 0.1mg/kg group by Tukey's *post hoc* test following One-way ANOVA (*P*<.001)
^ Indicates significantly different relative to 0.1mg/kg group by Dunnet's T3 *post hoc* test following Brown-Forsythe One-way ANOVA (*P*<.05)
* Indicates an apparent difference relative to VEH/CON by Dunnet's T3 *post hoc* test following Brown-Forsythe One-way ANOVA or Holm Sidak's or Tukey's *post hoc* test following One-way ANOVA (*P*<.05)
BAT, brown adipose tissue

Supplementary Statistical Results:

Figure 2: BDE congener analysis in liver of DE-71-exposed mouse dams and their adult female offspring. (**a**) F1 liver ∑PBDEs, Brown-Forsythe ANOVA: Exposure $F_{(2.0,3.5)}$=91.9, P<.001. F0 liver, One-way ANOVA: $F_{(2,8)}$ =17.8, P<.001. Liver ∑PBDEs (F1 vs F0), Mann Whitney U Test for F0 mdn= 3034 (2410-3721) and F1 mdn=208 (162-265) groups (U=0, P<.05). (**c**) F1 Liver BDE-28/33, One-way ANOVA: Exposure $F_{(2,9)}$=4.9, P<.05. F0 Liver BDE-28/33, Brown-Forsythe ANOVA: Exposure $F_{(2,4.6)}$=10.7, P<.05. F0 Liver BDE-66, One-way ANOVA: Exposure F(2,8)=3.3, n.s. F0 Liver BDE-154, One-way ANOVA: $F_{(2,8)}$=9.3, P<.01. F0 Liver BDE-183, $F_{(2,8)}$=12.7, P<.01. (**d**) F0 Liver BDE-47, One-way ANOVA: Exposure $F_{(2,8)}$=5.1, P<.05. Liver F0 BDE-85/155, One-way ANOVA: Exposure $F_{(2,8)}$=22.9, P<.001. Liver F0 BDE-99, One-way ANOVA: Exposure $F_{(2,8)}$=10.0, P<.01. Liver F0 BDE-100, One-way ANOVA: Exposure $F_{(2,8)}$=22.3, P<.001. Liver F1 BDE-153, Brown-Forsythe ANOVA, $F_{(2,3.4)}$=76.5, P<.01. Liver F0 BDE-153, One-way ANOVA: Exposure $F_{(2,8)}$=65.0, P<.0001. n=3-4 replicates/group, analyzed in triplicate. ∑PBDE, the sum of concentration of 9 (F0) or 2 (F1) PBDE congeners; F1, female offspring; F0, dams

Figure 3. DE-71 exposure produces elevated fasting blood glucose (FBG) and greater glucose intolerance in perinatally exposed female offspring but not than their mothers. (**a**) Offspring FBG, Two-way ANOVA: Time $F_{(1,50)}$=8.7, P<.01; Exposure $F_{(2,50)}$=5.0, P<.05; Interaction $F_{(2,50)}$=15.2, P<.0001. (**b**) Dams FBG, Two-way ANOVA: Time $F_{(1,49)}$=5.2, ns; Exposure $F_{(2,49)}$=0.6, ns; Interaction $F_{(2,49)}$=0.21, ns. n=7-12/group. ns, not significant

Figure 4. DE-71 exposure produces greater glucose intolerance after perinatal exposure compared to adult exposure. (**a**) Offspring IPGTT, RM Two-way ANOVA: Time $F_{(4,96)}$=110.7, P<.001; Exposure $F_{(2,24)}$=3.9, P<.01; Interaction $F_{(8,96)}$=2.7, P<.05. (**d**) Dams IPGTT, Mixed-effects model ANOVA (Geisser-Greenhouse's ε=0.55): Time $F_{(2.2,46.0)}$=90.8, P<.0001; Exposure $F_{(2,21)}$=1.5, n.s.; Interaction

$F_{(8,83)}$=2.0, n.s. (**b**) Mean values for integrated area under the IPGTT glucose curve ($AUC_{IPGTTglucose}$). Offspring $AUC_{IPGTTglucose}$, Brown-Forsythe ANOVA: Exposure $F_{(2.0,15.3)}$=4.9, P<.05. (**e**) Dams $AUC_{IPGTTglucose}$, Brown-Forsythe ANOVA: Exposure $F_{(2.0,14.9)}$=1.3, ns. (**c**) Offspring Latency to Maximum Glycemia, Brown-Forsythe ANOVA: Exposure $F_{(2,14.8)}$=3.7, P<.05. (**f**) Dams Latency to Maximum Glycemia, One-way ANOVA: Exposure $F_{(2,21)}$=1.6, n.s. (**g**) Offspring Percent basal IPGTT, RM Two-way ANOVA: Time $F_{(4,96)}$=83.6, P<.0001; Exposure $F_{(2,24)}$=3.2, P=.057; Interaction $F_{(8,96)}$=2.9, P<.05. (**i**) Dams Percent basal IPGTT, RM Two-way ANOVA: Time $F_{(4,80)}$=69.4, P<.0001; Exposure $F_{(2,20)}$=3.4, P=.06; Interaction $F_{(8,80)}$=1.6, n.s. (**h**) Offspring $AUC_{IPGTTglucose}$, One-way ANOVA: Exposure $F_{(2,24)}$=3.9, P<.05. (j) Dams $AUC_{IPGTTglucose}$, One-way ANOVA: Exposure $F_{(2,20)}$=3.3, P=.06. ns, not significant

Figure 5. DE-71 exposure causes less glycemia reduction and delayed glucose clearance after insulin challenge in female offspring (F1) but not (F0) their mothers. (**a**) Offspring ITT: RM Two-way ANOVA: Time $F_{(6,156)}$=62.1, P<.0001; Exposure $F_{(2,26)}$=7.4, P<.01; Interaction $F_{(12,156)}$=3.8, P<.0001. (**e**) Dams ITT, Mixed-model ANOVA: Time $F_{(6,165)}$=41.5, P<.0001; Exposure $F_{(2,28)}$=0.3, n.s.; Interaction $F_{(12,165)}$=1.0, n.s. (**b**) Offspring Inverse $AUC_{ITTglucose}$, One-way ANOVA: Exposure $F_{(2,26)}$=4.4, P<.05. (**f**) Dams Inverse $AUC_{ITTglucose}$, One-way ANOVA: Exposure $F_{(2,28)}$=0.01, n.s. (**c**) Offspring K_{ITT}, One-way ANOVA: $F_{(2,21)}$=3.1, P=.06. (**g**) Dams K_{ITT}, One-way ANOVA: $F_{(2,20)}$=1.1, n.s. (**d**) Offspring (F1) Latency to Minimum Glycemia, One-way ANOVA: $F_{(2,26)}$=8.5, P<.01. (**h**) Dams (F0) Latency to Minimum Glycemia, Brown-Forsythe ANOVA: $F_{(2,16.63)}$=1.7, n.s. (**i**) Offspring (F1) Percent basal ITT, RM Two-way ANOVA: Time $F_{(6,156)}$=71.4, P<.0001; Exposure $F_{(2,26)}$=2.4, n.s.; Interaction $F_{(12,156)}$=3.2, P<.0001. (**k**) Dams (F0) Percent Basal Glycemia, Mixed effects model ANOVA: Time $F_{(6,167)}$=38.0, P<.0001; Exposure $F_{(2,28)}$=0.2, n.s.; Interaction $F_{(12,167)}$=1.0, n.s. (**j**) Offspring $AUC_{ITTglucose}$, One-way ANOVA: Exposure $F_{(2,26)}$=3.1, P=.06. (**l**) Dams, $AUC_{ITTglucose}$, One-Way ANOVA: Exposure $F_{(2,27)}$=.21, n.s. Dunnet’s and Tukey’s *post-hoc* tests were used. ns, not significant

Figure 6. Endocrine-disrupting effects of DE-71 on glucose regulatory hormones in F0 and F1 female mice. (**a**) Offspring (F1) Plasma Insulin Levels, Kruskal-Wallis test: Exposure H(2)=10.5, *P*<.01. Dam (F0) Insulin Levels, Brown-Forsythe ANOVA: Exposure $F_{(2,41.5)}$=2.1, n.s. (**b**) Offspring Plasma Glucagon Levels, Brown-Forsythe ANOVA: Exposure $F_{(2,10.0)}$=3.7, *P*=.065. Dam (F0) Glucagon Plasma Levels, Brown-Forsythe ANOVA: Exposure $F_{(2,9.9)}$=3.0, *P*=0.1. (**c**) Offspring (F1) Plasma GLP-1 Levels: Exposure, One-way ANOVA: $F_{(2,30)}$=.6, n.s. Dam Plasma GLP-1 Levels, One-way ANOVA: Exposure $F_{(2,23)}$=6.0, *P*<.01 followed by Tukey's *post-hoc* test. Dunnet's, Dunn's and Tukey's *post hoc* tests were used. F1, female offspring; F0, dams; ns, not significant.

Figure 7. DE-71 exposure increases adrenal epinephrine content in F0 and F1 females and decreases brown adipose tissue mass in F1 female mice. (**a**) Adrenal Epinephrine Content. Offspring Epinephrine, Brown-Forsythe ANOVA: Exposure $F_{(2,9.3)}$=38.1, *P*<.0001. Dams Epinephrine, One-way ANOVA: Exposure $F_{(2,17)}$=16.3, *P*<.0001. (**b**) Intrascapular brown adipose tissue (BAT) normalized to body weight. Offspring BAT, One-way ANOVA: Exposure $F_{(2,57)}$=4.8, *P*<.05. Dam BAT: Brown-Forsythe ANOVA: Exposure $F_{(2,20.5)}$ =1.5, n.s. Dunnet's T3 or Tukey's *post-hoc* tests were used. F1, female offspring; F0, dams; BAT, brown adipose tissue; ns, not significant.

Figure 8. DE-71 exposure reduces hepatic activity of glutamate dehydrogenase (GDH) in F1 and F0 female mice. Offspring (F1) GDH, Brown-Forsythe ANOVA: Exposure $F_{(2,14)}$=59.1, *P*<.0001. Dams (F0) GDH, One-way ANOVA: Exposure $F_{(2,20)}$=46.1, *P*<.0001. F1, female offspring; F0, dams. Dunnett's T3 (F1) or Tukey's (F0) *post-hoc* tests were used.

Figure 9. DE-71 exposure increases hepatic levels of endocannabinoid (EC) and related fatty acid-ethanolamides in exposed F1 but not F0 female mice. **(a**) Offspring (F1) AEA, Brown-Forsythe ANOVA: Exposure $F_{(2,10.6)}$=4.1, *P*<.05. Offspring (F1) DHEA, Welch's ANOVA: Exposure $F_{(2,10.3)}$=6.0,

P<.05. Offspring OEA, Welch's ANOVA: Exposure $F_{(2,11.6)}$=3.4, *P*=0.069. (**b**) Offspring (F1) 2-AG, One-way ANOVA: Exposure $F_{(2,19)}$=.79, n.s. Offspring 2-DG, Brown-Forsythe ANOVA: Exposure $F_{(2,30)}$=1.15, n.s. (**c**) Dam (F0) AEA, One-way ANOVA: Exposure $F_{(2,9)}$=.64, n.s. Dam (F0) DHEA, One-way ANOVA: Exposure $F_{(2,8)}$=.16, n.s. Dam OEA, One-way ANOVA: Exposure $F_{(2,9)}$=.21, n.s. (**d**) Dam (F0) 2-AG, One-way ANOVA: Exposure $\mathrm{F}_{(2,9)}$=.81. Dam (F0) 2-DG, One-way ANOVA: Exposure $F_{(2,9)}$=.95, n.s. Dunnett's T3 and Tukey's *post-hoc* test was used. EC, endocannabinoid; AEA, arachidonoylethanolamide (Anandamide); DHEA, docosahexanoyl ethanolamide; OEA, n-oleoyl ethanolamide; 2-AG, 2-arachidonoyl-*sn*-glycerol; 2-DG, monoacylglycerol; 2-docosahexaenoyl-*sn*-glycerol. ns, not significant
